# Lactic acid production – producing microorganisms and substrates sources-state of art

**DOI:** 10.1016/j.heliyon.2020.e04974

**Published:** 2020-10-12

**Authors:** Elahe Abedi, Seyed Mohammad Bagher Hashemi

**Affiliations:** Department of Food Science and Technology, College of Agriculture, Fasa University, Fasa, Iran

**Keywords:** Biotechnology, Microbiology, Lactic acid, Fermentation, Microorganisms, Agricultural waste, Industrial waste

## Abstract

Lactic acid is an organic compound produced via fermentation by different microorganisms that are able to use different carbohydrate sources. Lactic acid bacteria are the main bacteria used to produce lactic acid and among these, *Lactobacillus* spp. have been showing interesting fermentation capacities. The use of *Bacillus* spp. revealed good possibilities to reduce the fermentative costs. Interestingly, lactic acid high productivity was achieved by *Corynebacterium glutamicum* and *E. coli,* mainly after engineering genetic modification. Fungi, like *Rhizopus* spp. can metabolize different renewable carbon resources, with advantageously amylolytic properties to produce lactic acid. Additionally, yeasts can tolerate environmental restrictions (for example acidic conditions), being the wild-type low lactic acid producers that have been improved by genetic manipulation. Microalgae and cyanobacteria, as photosynthetic microorganisms can be an alternative lactic acid producer without carbohydrate feed costs. For lactic acid production, it is necessary to have substrates in the fermentation medium. Different carbohydrate sources can be used, from plant waste as molasses, starchy, lignocellulosic materials as agricultural and forestry residues. Dairy waste also can be used by the addition of supplementary components with a nitrogen source.

## Teaser

Biotechnology; Microbiology; Lactic acid; fermentation; Microorganisms; agricultural; Industrial waste

## 1 Introduction

Lactic acid as an organic acid is authorized by the U.S. Food and Drug Administration as GRAS (generally regarded as safe). It provides leading roles in the food and non-food industry. i) It is utilized in the food industry including beverage industry (as food preservative, fermentation agent, acidulant, flavour enhancer, and decontaminant), antioxidant, prebiotic activity, cryoprotectant, viscosifier, ii) chemical industry mainly mosquito repellent, descaling agents, pH regulator, neutralizers, green solvent, cleaning agents, metal complexing agents, substitution of synthetic plastics derived from petro-chemically compounds and environmentally friendly alternative due to production of poly-lactic acid as biodegradable polymers for commercial uses such as fibers and films, production of propylene glycol, lactate esters, acrylic acid, propylene oxide, propanoic acidacetaldehyde, 2,3-pentanedione, and dilactide; iii) cosmetic industry as moisturizers, skin-lightening agents, skin rejuvenating agents, anti-acne agents, humectants, anti tartar agents; iv) medicine and pharmaceuticals industry as a building-block molecule, dialysis solution, mineral preparations, tablettings, prostheses, surgical sutures, controlled drug delivery system, immune-stimulant and manufacture of hygiene and esthetic products [1, 2]. Lactic acid is commonly sold as an 88% solution. The price varies with the application (e.g., food, pharmaceuticals, and PLA) and also depends on the price of commodity starch and sugar feedstocks used for fermentation. A range of around $3.0-$4.0/kg was reported in 2019 (https://www.pharmacompass.com). Upon annual growth of 16.2%, the global lactic acid market increased from 1,220.0 kilotons in 2016 to 1,960.1 kilotons in 2025. This should display USD 9.8 billion in the global market. Market studies mention that the major growth will be for medicines and cosmetics in the Latin America and the Asia Pacific region [2].

**Figure 1:**
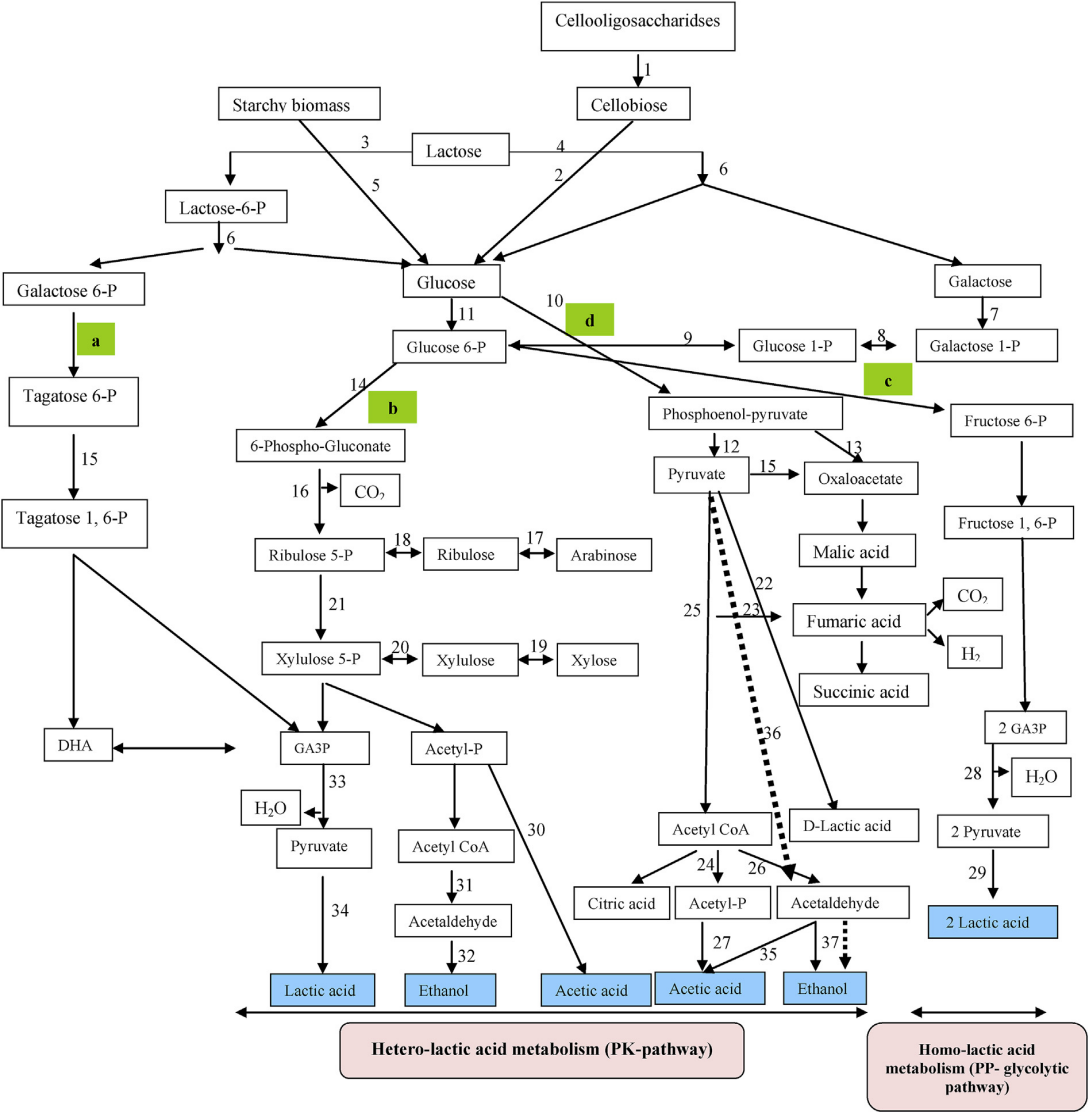
Pathways of lactic acid production from agro-industrial residues. Number on arrow catalyzed by enzyme and other reaction. 1: Exo β_1,4_ Glucanase, 2: β -Glucosidase, 3: lactose phosphotransferase system (*Lac*-PTS), 4: permease, 5: Amylase, 6: β-galactosidase, 7: ATP→ADP, 8: galactose-1-phosphate uridylyltransferase, 9: phosphoglucomutase, 10: NAD→ NADH, 11: ATP→ADP, 12: ATP→ADP, 13: Phosphoenolpyruvate carboxylase, 14: ATP→ADP, 15: ATP→ADP, 16: NAD^+^→NADH, 17: arabinose isomerase, 18: ribulokinase and ATP→ADP, 19: xylose reductase and xylitol dehydrogenase, 20: ATP→ADP, 21: ribulose 5-phosphate 3-epimerase, 22: D-lactic acid Dehydrogenase, 23: Pyruvate-fomarate lyase, 24: Pta, 25: Pyruvate dehydrogenase complex, 26: Aldehyde dehydrogenase, 2NADH→ 2NAD^+^, 27: Acetate kinase, 28: 4 ADP→ 4ATP, 2 NAD^+^→2NADH, 29: 2NADH→2NAD^+^, 30: ADP→ ATP, 31: NADH→ NAD^+^, 32: NADH→ NAD^+^, 33: 2ADP→ATP, NAD^+^→NADH, 34: Lactate dehydrogenase, NADH→NAD^+^, 35: Acetaldehyde dehydrogenase, 36: Pyruvate decarboxylase. 37: Alcohol dehydrogenase. GA3P: glyceraldehyde-3-P, DHAP: Dihydroxyacetone-P. A route: D-tagatose 6-phosphate pathway. B route: Pentose phosphoketolase (PK) pathway: for Hetero lactic acid metabolism. C route: Embden-Meyerhof-Parnas (EMP) pathway: for Homo lactic acid metabolism. D route: Glycolysis pathway in *E. coli*, *K. lactis* and *S. cerevisiae*.

The direct conversion of complex compounds to lactic acid can be categorized mainly into Four groups. a) The lactic acid producing fungi such as Rhizopus oryzae. b) amylolytic lactobacilli namely *Lb. amylovorous*, *Lb. manihotivorans*, *Lb. amylophilus* etc. c) The simultaneously degradation of substrate further treat with enzymes. d) glycolysis pathway in *E. coli*, *K. lactis* and *S. cerevisiae* [3, 4] (Figure 1).

The fermentation capacity by several LAB has been studied in order to produce LA. Plenty of lactic acid bacteria have amylase activity were originated from various plant and animal. Main obstructions lactic acid bacteria is that they require complex nutrients and slightly lower fermentation temperatures (˂ 45 °C) than other microorganism, which lead to increased costs and contamination risk and are also poor productivity due to the amylase production in the initial step, causing a long lag phase. Otherwise they require partially hydrolyzed substrates. Certain fungi including *Rhizopus sp*. can generate high content of lactic acid. They also specify with advantages compared with the bacterial process such as i) the consumption of a chemically defined medium (including inorganic nitrogen sources), which can facilitate product separation and purification, ii) consume both complex carbohydrates and pentose sugars iii) high product concentrations of pure L-lactic acid owing to metabolize high amount of glucose which is preferred for poly-lactide manufacture. For instance, fungal species of *R. oryzae* 2062 and *R. arrhizus* 36017 produce lactic acid in a single-stage simultaneous saccharification and fermentation process. In contrast, homofermentative lactic acid bacteria have highly more efficiencies than the fungi to convert sugars to lactic acid because production other byproducts such as ethanol and fumaric acid by *R. oryzae*-based process. Some researcher tried to enhance lactic acid production using a mutant of R. oryzae with declined alcohol dehydrogenase activity under oxygen limiting conditions. This strain generated almost 10-fold more lactic acid production when compared to the parent strain [3, 4]. *Bacillus spp*., allows reducing the LA production cost due to fewer nutrition demands and a high temperature of fermentation. Relatively to the use of fungi, the low LA productivity disadvantage of using wild-type yeasts can be overcome by engineering genetic modification [5]. Moreover, *Saccharomyces cerevisiae* is one of the more promising organisms that reveal high tolerance to low pH-values. Interestingly, good LA productivities were achieved by genetically modified *Candida* spp [5].

Relatively to substrate sources, worldwide there is a lot of interesting agro-industrial waste or sub-products with a lower value, which can be fermented by several organisms. Molasses, juices waste, starchy biomass, agricultural residues, and forestry residues that is rich in mono and disaccharides, which in some cases need to be hydrolysed by pectinases to enhance the LA production. To use dairy wastes as a substrate, mainly whey, it is necessary to use an enriched mediums, due to insufficient proteolytic enzyme activity [5, 6, 7, 8]. In this paper, different bacterial groups that capable of producing lactic acid at different rates and under different conditions were discussed.

In this paper, different bacterial groups that capable of producing lactic acid at different rates and under different conditions were discussed. Moreover, chemical and physical pretreatment of substrates were explained.

## 2 LA producing microorganisms

### 2.1 Bacteria

#### 2.1.1 Lactic acid bacteria

Lactic acid bacteria (LAB) are gram-positive microorganisms known as the main safe industrial-scale producers of lactic acid (LA). LA is produced by glycolysis pathway under anaerobic conditions, and this compound can be produced from hexoses and pentoses LAB metabolism pathways, as indicated in Figure 1. LA production yield and productivity depends on pH (3.5–9.6), temperature (5–45 °C), nutrients presence (such as amino acids, peptides, nucleotides and vitamins) and the LAB strain producers used (so far have been used strains belonging to the genus *Leuconostoc, Lactococcus, Lactobacillus, Pediococcus, Enterococcus, Streptococcus, Vagococcus, Aerococcus, Carnobacterium, Tetragenococcus*, *Oenococcus* and *Weissella*) [5, 6, 7, 8]. However, LAB species including *Lactobacillus, Lactococcus, Leuconostoc, Streptococcus,* and *Pediococcus* are also used as starter cultures in industrial food fermentations. Among LAB strains, *Lactobacillus* strains have great commercial importance due to high acid tolerance, high yield, and productivity, and can be engineered for the selective production of L/D-lactic acid [5]. However, there are some disadvantages when using the LAB for commercial LA production, such as the high requirement of complex nutrients (with increasing production costs) and the low fermentation temperature (that could result in contamination risks and prevention of simultaneous saccharification of starchy or cellulosic biomass and conversion to sugars by amylases enzymes and fermentation of sugars and lignocellulosic biomass) [9, 10]. However, the alkaliphilic LAB that includes *Marinilactibacillus*, *Halolactibacillus*, and *Alkalibacterium* spp. and other various strains from LAB genera, such as *Microbacterium* spp., *Enterococcus* spp., *Alkalibacterium* spp., *Exiguobacterium* spp., *Oceanobacillus* spp. and *Bacillus* spp., can produce LA at high pH-values (7.0–11.5), resulting in a contamination minimization during the fermentation process [9, 10, 11, 12]. For example, *Exiguobacterium* is a genus of bacilli, being the alkaliphile *Exiguobacterium* sp. strain 8-11-1 used to produce optically pure l-lactate, in nonsterile fed-batch fermentation with productivity of 8.15 g/L/h under glucose concentration of 80 g/L and using NaOH as a neutralizing agent [9].

Since the complex nutritional requirements of the LAB complicate industrial processes and enhance cost, genetic engineering methods by gene manipulation with plasmid transformation could improve the fermentation efficiency of LA production. Some microorganisms, such as *Corynebacterium glutamicum (section 1-3), Escherichia coli (section 1-4)* and yeasts lack activities for pyruvate-formate lyase and lactate dehydrogenase (LDH), and these genes can be inserted through gene sources of L-/D-LDH from LAB, bovine and fungi, to express the D(-)- LDH gene from LAB, producing D(-)-lactate in minimal medium with >99.9% optical purity.

Glucose fermentation by homofermentative LAB needs somewhat acidic to neutral pH. However, low pH, has an inhibitory impact on cellular metabolism, in turn lactic acid production. The large number of LAB cannot grow lower than pH 4. In order to maintain cell survival two solutions are used: i) lime is routinely introduced to the fermentors to keep a neutral pH, which cause to produce calcium lactate (>90% of the lactic acid). Subsequent fermentation, the broth containing calcium lactate would be acidified with sulfuric acid to generate lactic acid. High sulfuric acid consumption leads to form high content of insoluble calcium sulfate as gypsum compared to the amount of lactic acid produced, waste disposal concerns, further corrosion problems and a significant cost factor in the product recovery step of commercial operations. Ideally, microbial fermentation would take place in medium with a pH at or lower than the pK_a_ of lactic acid (the pK_a_ of lactic acid is 3.78), permitting direct purification of the acid form. ii) Metabolic engineering has been applied to modify for variants of Lactobacillus sp. with improved tolerance to the acidified medium generated during fermentation. Improved strains has been achieved after UV and nitrosoguanidine treatment, which they are capable to produce lactic acid at rates and yields like to those of the traditional, neutral-pH lactic acid processes. In order to maximize resistance to the acidic conditions inducing by lactic acid production, enzymes namely trehalose 6-phosphate phosphatase from *Propionibacterium freudenreichii* has been expressed in *Lb. lactis*, leading to 5- to 10-fold greater survivability at pH 3.0. Similarity, the enzymes in histidine decarboxylation pathway from *Streptococcus thermophilus* was expressed in Lb. lactis, making survival at pH levels as low as 3 in which the host cells were easily dying [1]. There are two fermentative LAB pathways:

A) The homofermentative LAB

LAB possesses the aldolase enzyme and can convert glucose almost exclusively into LA. The homofermentative LAB usually uses hexose and pentose sugars via the Embden-Meyerhof (by using glycolysis pathway and pentose phosphate pathway). Homofermentative LAB produces two LA molecules as a major end-product per mole of consumed glucose, with a theoretical yield of 1 g.g^−1^ and experimental yields among being this related to the type of the carbon source used [11]. For LA commercial production (more than 100 g/L of lactic acid) only homofermentative LAB is available due to the high yield (near maximal theoretical value), productivity and a high optical purity of lactic acid (>99%). Homofermentative LAB includes *Streptococcus*, *Lactococcus*, *Enterococcus*, *Pediococcus*, and some *Lactobacillus*, as shown in Table 1. Homofermentative *Lactobacillus* spp. includes mainly *Lb. delbruckii* subsp. *bulgaricus*, *Lb. acidophilus*, *Streptococcus salivarius* subsp. *thermophilus*, and *Lb. helveticus*. Abdel-Rahman et al. [13, 14] reported that *Enterococcus mundtii* QU 25 and engineered *Lactobacillus plantarum* could also metabolize homofermentative pentoses to LA.

B) The heterofermentative LAB

**Table 1:** Compilation of organisms studied for lactic acid (LA) production, with respective LA concentration, yield, productivity, substrate source and reference.

Organism	Lactic acid	Yield	Productivity	Source	Reference
	g/L	g/g	g/(L/h)		
**Homo and Heterofermentative LAB**					

*Lb. delbruckii* NCIMB 8130	90.0	0.97	3.8	Molasses	[125]
*Lb. delbrueckii* sp. *delbrueckii* ATCC 9649	58	0.48		Glucose	[13, 14]
*Lb. delbrueckii* sp. *lactis* ATCC 8000	83	0.83		Glucose	[13, 14]
*Lb. delbrueckii* sp. *lactis* DSM 20073	82	0.82		Glucose	[13, 14]
*Lb. delbrueckii mutant* DP3	77	0.64		Glucose	[13, 14]
*Lb. delbrueckii mutant* DP3, 19	68	0.57		Glucose	[13, 14]
*Lb. delbrueckii* sp. bulgaricus AU	20	0.45		Whey permeate	[13, 14]
*Lb. delbrueckii* sp. *bulgaricus* 5085	16	0.38		Whey permeate	[13, 14]
*Lb. delbrueckii* sp. *bulgaricus* 5085	7.9	0.18		Whey permeate	[13, 14]
*Lb. delbrueckii* sp. *bulgaricus* 5085	15	0.41	4	Whey permeate	[13, 14]
*Lb. delbrueckii* sp. *bulgaricus* ATCC 11842	-	-	-	Sorghum	[13, 14]
*Lb. delbrueckii* sp. lactis 447	55	0.85		Lignocellulose hydrolysate	[13, 14]
*Lb. delbrueckii* sp. *bulgaricus* 5085	7.9	0.18		Whey permeate	[13, 14]
*Lb. delbrueckii* sp. *bulgaricus* 5085	16	0.38		Whey permeate	[13, 14]
*Lb. delbrueckii* sp. *bulgaricus* CRL 870	12	-	-	Skim milk	[13, 14]
*Lb. delbrueckii* sp. *delbrueckii* ATCC 9649	106	0.82		Hydrolysate wheat flour	[13, 14]
*Lb. delbrueckii* IFO 3534	24	0.48		Hydrolysate newspaper	[13, 14]
	53	0.53		Hydrolysate pure cellulose	
*Lb. delbrueckii* sp. *bulgaricus* CBS 743.84	35	0.85		Glucose	[13, 14]
	37	0.82		Lactose	
*Lb. delbrueckii* sp. *bulgaricus* CNRZ 369	56	2.8		Glucose	[13, 14]
	32	1.6		Cellobiose	
	41	2.1		Xylose	
*Lb. delbrueckii* sp. *delbrueckii*	87	0.87		Glucose	[13, 14]
	94	0.94		Fructose + glucose	
	85	0.85		Sucrose	
*Lb. delbrueckii* sp. *delbrueckii* ATCC 9649	58	0.85		Glucose	[13, 14]
	40	0.75		Lactose	
*Lb. delbrueckii* sp. *bulgaricus* ATCC 11842	18	0.11		Hydrolysate of wheat flour	[13, 14]
	26	0.18		Hydrolysate wheat flour	
*Lb. delbrueckii* sp. *lactis* ATCC 12315	100	1.0		Hydrolysate potato	[13, 14]
	93	0.78		Hydrolysate potato waste	
*Lb. delbrueckii* IFO 3534	83	0.83		Glucose	[13, 14]
	55	0.55		Glucose	
*Lb. delbrueckii* MIX several strains	85	0.87		Hydrolysate maize + barley	[13, 14]
	71	0.73		Hydrolysate maize + barley	
*Lb. delbrueckii* NCIM-2365	90	0.9		Glucose	[13, 14]
	75	0.75		Glucose	
*Lb. delbrueckii* sp. *bulgaricus*	44	0.95		Whey	[13, 14]
	13	0.28		Whey	
*Lb. delbrueckii* sp. *bulgaricus* ATCC 11842	50	1.0		Whey	[13, 14]
	9.5	0.19		Whey	[13, 14]
*Lb. delbrueckii* sp. *bulgaricus* Ch H 2217	115	0.86		Whey	[13, 14]
*Lb. delbrueckii* sp. *bulgaricus* NRRL B-548	45	0.90		Lactose	[13, 14]
*Lb. delbrueckii* sp. *bulgaricus* ATCC 55163	50	0.64		Whey	[13, 14]
*Lb. delbrueckii* sp. *bulgaricus* ATCC 11842	-	-		Sorghum	[13, 14]
*Lb. delbrueckii* sp. *bulgaricus* CNRZ 369	25	0.48		Whey	[13, 14]
*Lb. delbrueckii* sp. *bulgaricus* NRRL B-548	52	0.58		Cellulose	[13, 14]
*Lb. delbreuckii*	35.4	0.35	0.75	Alfalfa fibers	[157]
*Lb. delbrueckii* NCIM 2025	81.9	0.94	1.36	Cassava bagasse	[164]
*Lb. delbrueckii* subsp. *delbrueckii* IFO 3202	28.0	0.28	0.78	Defatted rice bran	[13, 14]
*Lb. delbrueckii* mutant Uc-3	67.0	0.83	0.93	Sugarcane bagasse waste	[174]
*Lb.delbrueckii* ssp. *lactis* DSM 20073			9.9	Glucose	[24]
*Lb. delbrueckii* sp. *delbrueckii* ATCC 9649		0.82	1.6	Wheat	[13, 14]
*Lb. delbrueckii* sp. *bulgaricus* ATCC 11842		0.11	0.56	Wheat	[13, 14]
*Lb. delbrueckii* NCIM 2025			1.36	Cassava bagasse	[164]
**Homo and Heterofermentative LAB**					

*Lb. delbrueckii* ZU-S2		0.92	0.93–5.75	Corn cob residue	[206]
*Lb. delbrueckii* subsp*.delbrueckii* Mutant Uc-3		0.83	0.93	Sugarcane bagasse	[174]
*Lb. delbrueckii* UFV H2B20		0.99	0.82	Brewer's spent grain	[207]
*Lb. delbrueckii* NRRL B-445	108.0		0.9	Wood	[155]
*Lb. delbrueckii*	79	0.81	3.58	Broken rice	[208]
*Lb. delbrueckii*				Camel milk	[209]
*Lb. delbrueckii*				Cow milk	[209]
*Lb. delbrueckii*				Rice	[210]
*Lb. delbrueckii*				Grain cellulosic hydrolysate	[211]
*Lb. delbrueckii*	88			Molasses	[125]
*Lb. delbrueckii*				Yucca	[164]
*Lb. delbrueckii* sp. *delbrueckii*	83.45–93.28		1.57–3.7	Orange waste enzymatic hydrolysates	[216]
*Lb. delbrueckii* subsp. *delbrueckii* Mutant Uc-3	166		4.15	Molasses	[123]
*Lb. delbrueckii*	107	0.9	1.48	Sugarcane molasses, sugarcane juice and sugar beet juice	[13, 14]
*Lb*. *delbrueckii* spp. *delbrueckii*	4.2–6.72	0.94		Orange peel wastes hydrolysates	[212, 213]
*Lb. delbrueckii* and *B. amyloliquefaciens*	40	0.96	0.42	Cassava bagasse	[214]
*Lb. delbrueckii*	16.15	0.5	0.9	Cassava fibrous waste hydrolysis	[215]
*Lb .delbrueckii subsp. delbrueckii* NBRC3202	25.38	1.18	0.53	Kodo millet bran residue	[216]
*Lb. delbrueckii sp. bulgaricus* CICC21101	18			Corn stover	[217]
*Lb. delbrüeckii spp. bulgaricus*	26.56	0.540	0.553	Cheese whey	[177]
*Lb. helveticus* sp. *milano*	18	0.36		Glucose	[13, 14]
	42	0.84		Maltose	
*Lb. helveticus* ATCC 15009	17	0.38		Lactose	[13, 14]
	8.9	0.20		Whey	
*Lb. helveticus* Milano	40	0.83		Whey permeate	[13, 14]
*Lb. helveticus* sp. milano	44	-	-	Hydrolysate whey	[13, 14]
	41	-	-	Hydrolysate clarified whey	
	37	-	-	Whey, Ultrafiltration (UF)	
*Lb. helveticus* ATCC 15009	49	1.1		Whey	[13, 14]
*Lb. helveticus* L89				Whey	[13, 14]
*Lb. helveticus* ATCC 15009	65.5	0.66	2.7	Cheese whey	[218]
*Lb. helveticus*	10.1	0.23	5.1	Cheese whey	[219]
*Lb. helveticus* NCDO 1844	47	1.2		Cheese Whey	[13, 14]
*Lb. helveticus* R211	38.0	-	19–22	Cheese whey	[218]
*Lb. helveticus*			10.5	Cheese whey	[218, 219, 220]
*Lb. helveticus* R211	66.0		1.4	Cheese whey	[13, 14]
*Lb. helveticus*&*K. marxianus, Lb. helveticus* (mixed culture)	15.5	0.45	10.0	Cheese whey	[219]
*Lb. helveticus*&*Lb. bulgaricus* (mixed culture)	14.6	0.35	9.4	Cheese whey	[219]
*Lb. helveticus*&*Lb. bulgaricus*& K. marxianus (mixed culture)	19.8	0.47	12.8	Cheese whey	[219]
*Lb. rhamnosus* ATCC 10863	68.0	0.76		Glucose	[13, 14]
Lb. rhamnosusATCC 7469	28	0.93		Glucose	
Lb. rhamnosusDSM 20024	22	0.74		Glucose	
*Lb. rhamnosus* ATCC 7469	24	0.80		Glucose	
*Lb. rhamnosus* CCM 1753	37	0.74		Lignocellulose hydrolysate	
*Lb. rhamnosus* ATCC 7469	18	0.40		Molasses	
*Lb. rhamnosus* ATCC 7469	30	0.71		Whey permeate	
*Lb. rhamnosus* ATCC 10863	30	0.71		Whey permeate	
*Lb. rhamnosus* ATCC 7469	21	0.38		Lactose	
*Lb. rhamnosus* ATCC 10863	17	0.86		Glucose	
	14	0.71		Fructose	
	16	0.81		Glucose + fructose	
	15	0.73		Sucrose	
*Lb. rhamnosus* ATCC 10863	45	-	-	Alpha-cellulose	
**Homo and Heterofermentative LAB**					

	28			Switch grass cellulose	
*Lb. rhamnosus* ATCC 10863	16	0.81		Hydrolysate molasses	
*Lb. rhamnosus* ATCC 10863	58	0.95		Glucose	
*Lb. rhamnosus* ATCC 10863	29	1.00		Hydrolysate wood	
*Lb. rhamnosus* ATCC 11443	53	0.66		Glucose	
*Lb. rhamnosus* ATCC 7469	34	1.1		Glucose	
*Lb. rhamnosus* ATCC 10863	80	0.74		Sucrose	
	80	0.89		Glucose	
	38	0.76		Glucose	
	32	0.80		Glucose	
	79	0.79		Glucose	
	25	0.91		Glucose	
	771	-		Glucose	
	45			Cellulose	
*Lb. rhamnosus* ATCC 9595 (CECT288)	32.5	0.88	5.41	Apple pomace	[13, 14]
*Lb. rhamnosus* CECT-288	42.0	0.38	0.87	Cellulosic biosludge	[170]
*Lb. rhamnosus* ATCC 7469	73.0	0.97	2.9	Paper sludge	[175]
*Lb. rhamnosus* ATCC 10863	67	0.84	2.5	Glucose	[13, 14]
*Lb.rhamnosus* IFO 3863		0.53–0.77	2.90–13.15	Glucose	[221]
*Lb. rhamnosus* ATCC 9595 (CECT288)		0.36–0.88	0.82–5.41	Apple pomace, cellulosic biosludge	[13, 14]
*Lb. rhamnosus* ATCC 7469		0.97	2.9	Paper sludge	[175]
*Lb. rhamnosus* and *Lb. brevis* (mixed culture)	20.95	0.70	0.58	Corn stover	[122]
*Lb. rhamnosus* ATCC 7469	18.58	0.73	–	Liquid distillery stillage	[222]
*Lb. rhamnosus* LA-04-1	82	0.81	3.73	White rice bran hydrolysate	[223]
*Lb. rhamnosus* ATCC 7469	34.7	0.81	0.66	Liquid distillery stillage	[222]
	42.2	0.99	1.22	Liquid distillery stillage	[222]
*Lb. rhamnosus*				Date juice	[133]
*Lb.rhamnosus*				Glucose	[224]
*Lb. rhamnosus* ATCC 7469	73.2–179	0.81	0.76	Recycled paper sludge	[225]
*Lb. rhamnosus* ATCC-10863	60			Softwood pre-hydrolysate and paper mill sludge	[226]
*Lb. rhamnosus*	41.65	0.83	0.87	Cassava wastewater	[227]
*L. rhamnosus* ATCC 7469	97.1		1.80	Bread stillage	[200]
*Lb.rhamnosus* HG09F5-27	157.22		8.77	Yam tuber starch	[228]
*Lb rhamnosus* 6003	45.5			Food waste	[229]
*Lb. rhamnosus*	22–40	76.9	1.22	Solid carob waste	[230]
*Lb. rhamnosus* PCM 489	27.5			Cheese industry – whey	[231]
*Lb. rhamnosus* B103	143.7			Dairy industry waste	[232]
*L. rhamnosus* ATCC 7469	58.01		1.19	Brewer's spent grain	[233]
*Lb. bulgaricus* NRRL B-548	38.7	0.90	3.5	Lactose, glucose, and galactose	[234]
*Lb. bulgaricus* ATCC 8001, PTCC 1332	24.6	0.81	-	Cheese whey	[235]
*Lb. bulgaricus* CGMCC 1.6970	70.70–113.18		1.47–2.36	Cheese whey powder	[236]
*Lb. bulgaricus*	19.5		1.22	Cheese whey	[182]
*Lb. bulgaricus* & *K. marxianus* (mixed culture)	16.2	0.41	10.5	Cheese whey	[13, 14]
*Lb. casei* NRRL B-441	82.0	0.91	5.6	Glucose	[13, 14]
	120	0.67	-	Hydrolysate barley flour	[13, 14]
*Lb. casei* SU No 22	16	0.32		Whey	[13, 14]
	20	0.39		Deproteinised whey	[13, 14]
*Lb. casei* NRRL B-441	112	0.68		Liquefied barley starch + glucoamylase	[13, 14]
	162	0.87		Liquefied barley starch + glucoamylase + alpha-amylase	
	36	0.20		Barley flour	
*Lb. casei* L100	50	0.83		Corn starch	[13, 14]
*Lb. casei Shirota*	94 82.6	0.92 2.5	2.61 2.50	Mixed food waste bakery waste	[237]
*Lb.casei* CICC 6056	55.1	0.835	0.574	Sophora flavescens residues	[238]
*Lb.casei*	21.3		0.63	Sugarcane bagasse	[239]
*Lb. casei* SU No 22	45	0.45	2.0	Whey	[13, 14]
**Homo and Heterofermentative LAB**					

*Lb. casei*	22	0.44		Whey	[13, 14]
*Lb. casei* NRRL B-441	80	0.89		Glucose	[13, 14]
*Lb. casei*	-	0.10	0.13	Banana wastes	[168]
*Lb. casei*	39.1–63.3	0.51–0.91		Food waste (mango, orange, green peas and)	[240]
*Lb. casei* subsp. *rhamnosus* NRRL-B445 and *Lc. lactis* subsp. *lactis* ATCC19435	60.3	-	3.20	Date juice	[133]
*Lb. casei* ATCC 10863	44	0.44	1.22	Ram horn hydrolysate	[241]
*Lb. casei* NRRL B-441	96.0	0.93	2.2	Cheese whey	[182]
*Lb. casei* SU No. 22 and *Lb. lactis* WS 1042 (mixed culture)	22.5	0.48	0.93	Cheese whey	[13, 14]
*Lb.casei* subsp. *casei* CRL 686			0.97	Glucose	[13, 14]
*Lb.casei* NRRL B-441		0.74–1	3.5–5.6	Glucose	[242]
*Lb.casei* LA-04-1		0.90	2.14	Glucose	[242]
*Lb. casei* NRRL B-441		0.93	2.5–3.97	Cheese whey	[13, 14]
*Lb. casei* NCIMB 3254			1.40	Cassava bagasse	[164]
*Lb. casei* NRRL B-441	162.0		3.4	Barley	[13, 14]
*Lb. casei*	33.73			Whey	[13, 14, 243]
*Lb. casei*				Molasses	[148]
*Lb. casei* A-8	~130			Reuse of anaerobic digestion effluent	[244]
*L. casei*				Yucca	[164]
*Lb. casei* M-15				Molasses	[129]
*Lb. lactis* ATCC 4797	12.5–24.3			Casein whey permeate	[245]
*L. lactis*				Molasses	[246]
*L. lactis*				Pineapples syrup	[246]
*L. lactis* WS 1042	11	0.22		Whey	[13, 14, 243]
*L. lactis* sp. *lactis* 2432	8.3	0.21		Whey permeate	
*L. lactis* sp. *cremoris* 2487	37	0.88	4.6	Whey permeate	
*L. lactis* sp. *lactis* 5085	37	0.88		Whey permeate	
*L. lactis* WS 1042	15	0.30		Deproteinised whey	
*L. lactis* sp. *lactis* 2432	9.0	0.20		Whey permeate	
*L. lactis* sp. *cremoris* SBT 1306	80	1.5		Lactose	
*L. lactis* sp. lactis ATCC 19435	96	0.76		Hydrolysate wheat flour	
*L. lactis* sp. *lactis* AS211	95	0.77		Hydrolysate wheat flour	
*L. lactis* sp. *lactis* NRRL B-4449	6.6	0.16		Waste paper	
*L. lactis* IO-l JCM 7638	23	0.45		Xylose	
	28	0.70		Xylose + glucose	
*L. lactis* sp. *lactis* ATCC 13673	36	1.0		Glucose	
	13	0.42		Xylose	
*L. lactis* sp. *lactis* ATCC 19435	4.9	0.86		Glucose	
	3.2	0.70		Maltose	
*L. lactis* sp. *lactis* NRRL B-4449	6.6	0.66		Glucose	
	2.8	0.28		Galactose	
	5.8	0.58		Mannose	
	1.8	0.18		Xylose	
		0.16		Hydrolysate cellulose + glucose + mannose + xylose + galactose	
*L. lactis* IFO 12007 + *Aspergillus awamori* IFO 4033	25	0.50		Potato starch	[13, 14]
*L. lactis* IO-l JCM 7638	24	0.96		Glucose	
*L. lactis* sp. *lactis* AS211	107	0.91		Hydrolysate wheat flour	
*L. lactis* sp. *lactis* ATCC 19435	106	0.88		Hydrolysate wheat flour	
*L. lactis* sp. *lactis* ATCC 19435	90	0.98		Hydrolysate wheat flour	
	75	1.0		Un hydrolysate wheat flour + glucose	
	53			Hydrolysate wheat flour	
*L. lactis* 65.1	39	0.75		Glucose	
*L. lactis* IFO 12007	25	0.50		Potato starch	
*L. lactis* sp. *lactis* ATCC 19435	65	1.5		Glucose	
*L. lactis* sp. *lactis* ATCC 19435	0.3	0.3		Glucose	
*L. lactis* IO-l JCM 7638	45	0.90		Glucose	
**Homo and Heterofermentative LAB**					

*L. lactis* 65.1	5.7	1.1		Glucose	
*L. lactis* IO-l JCM 7638	45	0.90		Glucose	
	66	0.88		Glucose	
*L. lactis* sp. *lactis* ATCC 19435	5.4	0.92		Glucose	[149]
	5.1	1.0		Maltose	
	96	0.76		Hydrolysate wheat flour	[13, 14]
*L. lactis* sp. *lactis biovar diacetylactis* CNRZ 2125	38	0.73		Lactose + citrate	
*L.lactis* BME5-18 M		0.97	2.2	Glucose	[83]
*L. lactis* IO-1			4.5	Glucose	[247]
*L. lactis* sp. *lactis*					
ATCC 19435		0.76	3.0	Wheat	[13, 14]
*L. lacti*s sp. *lactis*					
IFO 12007		0.76	0.6	Cassava	[248]
*L. lactis* sp. *lactis*					
AS211		0.77	1.7	Wheat	[13, 14]
*L. lactis* ATCC19435	92.5	0.68	0.5	Artichoke hydrolysate	[249]
*L. lactis* IL 1403/pCUSαA	15.6	0.89	1.57	Soluble starch	[13, 14]
*L. lactis* IO-1	10.9	0.36	0.17	Sugar cane baggage	[165]
*Lb. lactis* ssp. *lactis* IFO 12007	90.0	0.76	1.6		[248]
*Lb. lactis* NCIM 2368	17.01–72.24			Glucose	[250]
*Lb. plantarum* NRRL B-787	17	0.42		Solid waste	[13, 14]
*Lb. plantarum* NRRL B-788	19	0.46		Solid waste	
*Lb. plantarum* NRRL B-813	18	0.43		Solid waste	
*Lb. plantarum* NRRL B-531	18	0.43		Solid waste	
*Lb. plantarum*	17	0.70		Corn syrup	[13, 14]
Engineered *Lb. plantarum* NCIMB 8826 (GMO)	73.2–141.9	0.9–0.93	2.95	Glucose and xylose	[251]
*Lb. plantarum*	15	0.30		Hydrolysate soluble starch	[13, 14]
*Lb. plantarum*	15	0.30		Hydrolysate tapioca starch	
*Lb. plantarum* NRRL B-531	5.4	0.54		Glucose	[13, 14]
	3.7	0.37		Galactose	
	5.7	0.57		Mannose	
		0.43		Hydrolysate cellulose: glucose, mannose, xylose, galactose	
*Lb. plantarum* NRRL B-787	6.2	0.62		Glucose	
	4.0	0.40		Galactose	
	6.6	0.66		Mannose	
		0.42		Hydrolysate cellulose: glucose, mannose, xylose, galactose	
*Lb. plantarum* NRRL B-788	6.0	0.60		Glucose	
	4.9	0.49		Galactose	
		0.46		Hydrolysate cellulose: glucose, mannose, xylose, galactose	
*Lb. plantarum* NRRL B-813	7.3	0.73		Glucose	
	4.7	0.47		Galactose	
	8.3	0.83		Mannose	
		0.43		Hydrolysate cellulose: glucose, mannose, xylose, galactose	
*Lb. plantarum* USDA 422	5.2	0.52		Glucose	
	3.1	0.31		Galactose	
	6.2	0.62		Mannose	
	1.3	0.13		Xylose	
*Lb. plantarum*	46.4	0.46	0.64	Alfalfa fibers	[252]
*Lb paracasei* (NBRC 15889)	~100			Brown rice polish	[161]
*Lb*.*uvarum*	139.71				
*Lb farraginis* (NRIC 0676)	~125				
*Lb brevis*	160.97				
*Lb plantarum* (WCFS1)	137.67				
**Homo and Heterofermentative LAB**					

*Lb plantarum* (JCM 1149)	~115				
*Lb. plantarum* A6	8.41	0.98	-	Mussel processing wastes	[13, 14]
*Lb. plantarum* ATCC 21028	41.0	0.97	1.0	Synthetic lactose medium	[13, 14]
*Lb. plantarum* NCIMB 8826	73.2	0.85	3.86	Corn starch	[253]
*Lb. plantarum*				Bamboo	[254]
*Lb. plantarum* A6	86.6	0.89	4.54	Glucose	[255]
*Lb. plantarum* ΔldhL1	73.2	0.85	3.86	Raw starch	[255]
*Lb. plantarum* ΔldhL1/pCU-CelA	1.27	-	-	Cellohexaose	[253]
*Lb. plantarum* ΔldhL1/pCU-CelA	1.47	-	-	β-glucan	
*Lb. plantarum* ΔldhL1-xpk1:tkt	38.6	0.82	3.78	Arabinose	
*Lb. plantarum*ΔldhL1-xpk1: tkt-Δxpk2/pCU-PXylAB	41.2	0.89	1.60	Xylose	
Engineered *Lb. Plantarum* NCIMB 8826 (GMO)	55.2–102.3	0.879	1.77–2.61	Hardwood pulp, barley extract	[256]
*Lb. plantarum*	28.45–34.19 39.72–42.34	0.87–0.94 0.93–0.99	4.57–14.22 7.56–9.93	Glucose Hydrolysate of microalga Chlorella vulgaris ESP-31	[257]
*Lb plantarum* BP04	57.5			Dining-hall food waste	[201]
*Lb. plantarum*	117.1		0.81	Brown rice	[258]
*Lb. plantarum* DldhL1: PxylABxpk1: tkt-Dxpk2: PxylAB	39.7–74.2	0.78–0.79	1.53–2.85	Glucose/xylose mixture	[259]
*Lb. plantarum* NCDC 414				Vegetable juices	[260]
*Lb. amylovorus* ATCC 33620	4.2		0.1	Potato	[140]
*Lb. amylophilus* GV6	76.2	0.70	0.8		[146]
*Lb. amylovorus* ATCC 33622	93	0.52		Hydrolysate barley flour	[13, 14]
*Lb. amylophilus* ATCC 49845	21	0.95		Glucose	
	33	0.73		Hydrolysate corn starch	
*Lb. amylovorus* ATCC 33620	4.8	0.48		Cassava starch	
	10	1.0		Corn starch	
	4.2	0.42		Potato starch	
	7.9	0.79		Rice starch	
	7.8	0.78		Wheat starch	
*Lb. amylovorus* ATCC 33622	45	0.82		Raw corn starch	
*Lb. amylovorus* NRRL B-4542	114	0.63		Barley flour + gluco amylase	
*Lb. amylophilus* ATCC 49845	-	-		Glucose	
*Lb. amylophilus* ATCC 49845	30	0.60		Starch	
*Lb. amylophilus* GV6	27.3		0.3	Barley	
*Lb. amylophilus* BCRC 14055	21.62	0.98	0.31	Starch	[261]
*Lb. amylophilus*				Corn	[146]
*Lb. amylophilus*				Potato	[146]
*Lb. amylophilus*				Wheat (bran or flour)	[143]
*Lb. zeae* ATCC 393	21	0.71		Glucose	[13, 14]
*Lb. zeae* ATCC 393	37	0.98	5.0	Glucose	
*Lb. salivarius* sp. *salivarius* ATCC 11742	28	0.92		Glucose	
*Str. thermophilus*	18	0.50		Whey permeate	
*Str. thermophilus*	15	0.35		Whey permeate	
*Str. thermophilus*	19	0.47		Whey permeate	
*Str. thermophilus* CRL 807	8.5			Skim milk	
*Str. thermophilus*	40			Lactose	
*Str. thermophilus*	24.18–39.71		0.55–0.80	Magazine and office paper	[262]
*Lb. coryniformis* ssp. *torquens* ATCC 25600	24.0		0.5	Cellulose	[154]
*Lb. coryniformis ssp torquens* ATCC					
25600	23.1	0.51	0.48	Cardboard waste	[154]
*Lb. coryniformis* ssp. *torquens* ATCC 25600	39	0.98	2.6	Glucose	[13, 14]
*Lb. coryniformis* *Lb paracasei*	91.6–97.1	0.91–0.96	2.08–2.7	Curcuma longa waste (food waste)	[263]
*Lb. coryniformis* subsp. torquens	57.0	0.97	2.8	Pulp mill residue	[264]
*Lb. coryniformis* sub*. Torquens* ATCC 25600	36.6	0.46	1.02	Hydrodictyon reticulum	[199]
*Lb. coryniformis* sp. *torquens* ATCC 25600	23.4	0.51	0.49	Waste cardboard	[154]
*Lb. kefir*	9.8	0.20		Paneer whey	[13, 14]
*Lb. acidophilus* R	8.6	0.17		Paneer whey	
**Homo and Heterofermentative LAB**					

*Lb. acidophilus* CRL 640	14			Skim milk	[13, 14]
*E. faecium*	11	0.45		Hydrolysate cod + corn syrup	[13, 14]
*E. faecium*	27	0.91		Alfalfa	[13, 14]
*E. faecalis* RKY1	144.0	0.96	3.56–6.20	Glucose	[136, 265]
*E. faecium* No. 78			3.04	Sago	[266]
*E. faecalis* RKY1		0.93–1.04	0.5–4.8	Corn, wheat, tapioca, potato	[136, 267]
*E. faecalis* RKY1			1.7	Wood	[268]
*E. faecalis* QU 11	55.3	0.991		Glycerol	[269]
*E. faecalis* RKY1	95.7		4.0	Molasses	[140]
*E. faecalis* RKY1	93.0		1.7	Wood	[140]
*E. faecium* No. 78	36.3	0.57	1.96	Liquefied sago starch	[270]
*E. faecalis* RKY1	92–94	–	6.03–6.2	Glucose	[136]
*E. faecalis* RKY1	48.0	0.92	4.0	Wood hydrolyzate	[271]
*E. durans* BP130	28.8	0.85	0.24	Food waste	[12]
*E. mundtii* QU 25	67.2–129	0.78–0.90	0.76–1.2	Glucose/xylose mixture	[272]
*E. faecium* strain FW26	33.3	0.84		Banana peels and food wastes mixture	[273]
*Ped. acidilacti*	13	0.51		Hydrolysate cod + corn syrup	[13, 14]
Engineered Pediococcus acidilactici	87.8–104.5		1.22–1.45	Corn stover feedstock	[236]
*Lb. plantarum* NRRL					
B-4496, *Lb*. *acidophilus* NRRL B-4495, and *L*. *reuteri* B-14171				Egg white hydrolysates	[274]
*Lb. manihotivorans* LMG18011	48.7	0.098	0.76	Food wastes	[162]
*Lb. pentosus* NRRL B-227	21	0.51		Solid waste	[13, 14]
*Lb. pentosus* NRRL B-473	18	0.43		Solid waste	
*Lb. pentosus*	46	0.92		Glucose	
	27	0.54		Xylose	
	90	1.8		Glucose + xylose	
	40	0.70		Hydrolysate wood	
*Lb. pentosus* NRRL B-473	6.9	0.69		Glucose	
	5.9	0.59		Galactose	
	7.4	0.74		Mannose	
	1.4	0.14		Xylose	
		0.43		Hydrolysate cellulose: glucose + xylose + mannose + galactose	
*Lb. pentosus* ATCC 8041	21.8	0.77	0.84	Vine-trimming wastes	[163]
*Lb. sakei* KTU05-06, *Pediococcus acidilactici +* KTU05-7 + *P. pentosaceus* KTU05-9	40.0–93.0	0.62–1.45	0.83–1.94	Wheat bran	[275]
	28.4–54.6	0.50–0.97	0.59–1.14	Spent distiller's grain with solids	
	11.3–33.4	0.33–0.98	0.24–0.70	Brewer's spent grain	
*Lb. pentosus* ATCC-8041	23.0	0.93	0.45	Nannochloropsis salina	[110]
*Lb. pentosus* CHCC 2355		0.88		Wheat straw	[158]
*Lb. pentosus* ATCC 8041		0.65–0.77	0.1–0.9	Vine-trimming wastes/Corn Stover	[152, 158]
*Lb. pentosus*				Grape marc	[276]
*Lb. pentosus*				Wheat straw	[158]
*Lb. pentosus* CECT4023T	21	0.48–0.7		Gardening lignocellulosic residues	[277]
*Lb. pentosus* CECT-4023T (ATCC-8041)	46	0.78	0.933	Hemicellulosic hydrolyzates from trimming wastes of vine shoots	[278]
*Lb. paracasei* LA1	23.4	0.72	0.23	Wastewater sludge	[176]
*Lb. paracasei* LA104	37.11	0.46	1.03	*Hydrodictyon reticulum*	[199]
*Lb. paracasei* No. 8	81.5		2.7	Sweet sorghum	[13, 14]
*Lb. paracasei* No. 8	84.5		2.4	Rye	[13, 14]
*Lb. paracasei* No. 8	106.0		3.5	Sweet sorghum	[13, 14]
*Lb. paracasei* NCBIO01-M2	223.7		5.53	Glucose	[279]
*Lb.paracasei*	169.9		1.42	Molasses enriched potato stillage	[280]
*Lb. paracasei* DSM 23505	123.7	0.91		Chicory flour	[281]
*L. paracasei* A-22	80.10	0.97	1.48	Agro-industrial waste such as sunflower seed hull, brewers' spent grain, and sugar beet pulp	[282]
*Lb. paracasei* subsp. paracasei CHB2121	192	0.96	3.99	Glucose	[283]
*Lb. paracasei* KCTC13169	92.5	0.9	8 1.2	Artichoke tuber extract	[284]
*Lb.* sp. RKY2	129.0		2.9	Rice	[140]
**Homo and Heterofermentative LAB**					

*Lb.* sp. RKY2			3.1	Rice and wheat bran	[140]
*Lb.*sp. strains A28a	~52.4	0.07	0.27	Mixed food waste	[285]
		0.22	0.27	Starch	
		0.14	0.27	Sugar	
*Lb.*sp. strains A59		0.14	0.53	Mixed food waste	
		0.43	0.53	Starch	
		0.29	0.53	Sugar	
*Lb.*sp. strains A211		0.14	0.37	Mixed food waste	
		0.41	0.37	Starch	
		0.24	0.37	Sugar	
*Lb. brevis* ATCC 14869	12.5	0.57	0.56	Glucose, xylose or a glucose/xylose mixture	[286]
*Lb. rhamnosus + L. brevis (mixed culture)*	14.8	0.73	0.4	Glucose/xylose mixture	[287]
*Lb. brevis*	15	0.22		Cottonseed cake, wheat straw, sugarcane bagasse	[288]
	10	0.49			
	12.5	0.52			
*Lb. brevis and Lb. plantarum*	~15–35	0.52–0.8		Lignocellulosic biomass	[289]
*Lb. brevis* CHCC 2097 and *Lb. pentosus* CHCC 2355	7.1	0.95	-	Wheat straw	[158]
*Exiguobacterium* sp. strain 8-11-1	-	-	8.15		[290]
*Lb. bifermentans* DSM 20003		0.83	1.17	Wheat straw	[159]
*Halolactibacillus halophilus* JCM 21694	65.8	0.83	1.1	Sucrose	[291]
*Lb.* sp. G-02 and *Aspergillus niger* SL-09 (mixed culture)	120.5	0.95	3.3	Artichoke tubers	[91]
*Sporolactobacillus* sp. strain CASD	207	0.93	3.8	Peanut meal and glucose	[28]
*Sporolactobacillus inulinus* YBS1-5	107.2	0.85	1.19	Corncob residues & cottonseed meal	[292]
*Sporolactobacillus inulinus* YBS1-5	87.3–99.5	0.65–0.89	0.81–1.94	Wheat bran	[293]
*Sporolactobacillus* sp. strain CASD	82.8	0.94	1.72	Glucose	[40]
*Sporolactobacillus inulinus*	93.4		1.37	Glucose	[294]
*Sporolactobacillus inulinus* YBS1-5	70.5		0.65	Corn stover	[295]
*Sporolactobacillus laevolacticus* DSM442	144.4		4.13	Cotton seed	[296]
*Lb. sp. G-02*	141.5	0.94	4.7	Artichoke tubers	[297]
*Lb*. sp. RKY2	94.06	0.98	1.06	Cheese whey	[184]
*Lb.* TY50	36.29	ND		Kitchen waste	[298]
*Lb. sp.*	23.21			Food waste + cu^+2^	[201]
*Lactobacillus* sp. B2	19.5L		0.81	Crustacean waste	[299]
*Lb. paracasei* ATCC 334	1.2		1	Chlorella	[300]
*Lb. lactis subsp. lactis* NBRC 12007	0.8		1		
*Lb. reuteri* JCM 1112	1.02–4.29			Glucose-sucrose	[301]
Lactococcus lactis JCM 7638				Glucose-sucrose	
*Lb. gasseri* NCIMB 11718	8.42–18.7			Glucose-sucrose	
*Lb. plantarum* NCIMB 8826				Glucose-sucrose	
*Lb. paracasei* ATCC 334	8.01–12.3			Glucose-sucrose	
	5.17–7.03				
	7.77–9.60				
*Lb. paracasei* 7B	52.61	0.96	2.25–3.23	Wood ligonocellulosic hydrolysate	[302]
*Lb. paracasei* h601	21.19				
*Lb. plantarum* A1	41.91				
*Lb. plantarum* K1	25.22				
*Lb. plantarum* N14-2	36.95				
*Lb. fermentum* h602	31.11				
*Lb. fermentum* ATCC 14931	12.99				
*Lb. fermentum* E1	5.91				
*Lb. brevis* ATCC 8287	39.15				
*B. coagulans* T10-2	13.44				
*B. coagulans* T5-1	4.43				
W. paramesenteroides H1-6	18.49				
**Homo and Heterofermentative LAB**					

*Lb. points* (32%), *Lb. frumenti* (10%), *Lb. acidophilus* (8%), *Lb. amylovorus* and *Bifidobacterium (mixed culture)*	10–20			Acidogenic fermentation of fruit and vegetable wastes	[303]
*Lb plantarum + Lb buchneri, + Lb rhamnosus; Lb. plantarum + Lb paracasei*	30.4–127.9			Maize and amaranth	[304]
*Lb. manihotivorans* LMG18011	48.7	1.11		Starch and food waste	[162]
*Lb. rhamnosus & B.coagulans*	112.5	0.88	2.74	Cassava bagasse	[305]
*Lb. delbrüeckii spp. bulgaricus*	31.70	0.645	0.660	Hydrolysed cheese whey	[177, 275]
*P. acidilactici* KTU05-7	24.54	0.499	0.511		
*P. pentosaceus* KTU05-8	21.45	0.396	0.447		
*P. pentosaceus* KTU05-9	25.49	0.519	0.531		
*P. pentosaceus* KTU05-10	19.46	0.396	0.405		
*P. acidilactici* KTU05-7	27.86	0.567	0.580		
*P. pentosaceus* KTU05-8	25.21	0.513	0.525		
P. *pentosaceus* KTU05-9	28.06	0.571	0.584		
*P. pentosaceus* KTU05-10	22.82	0.464	0.475		
*P. acidilactici*	97.3		0.95	Corn stover	[306]
*P. acidilactici* ZP26	77.66		1.06	Corn stover	[307]
*Pediococcus acidilactici* (DSM, 20284)	~125			Brown rice polish	[161]
*Pediococcus pentosaceus* (ATCC 25745)	~65				
*Lb. buchneri* NRRL B-30929	13.35			Elephant grass	[308]
*E. casseliflavus*/*Lb. casei* (mixed culture)	95	0.63	0.49	Glucose/xylose mixture	[309]
*Actinobacillus succinogenes*	183.4	0.97	1.53	Glucose	[310]
*Pediococcus acidilactici* TM14 *and Weissella paramesenteroides* TA15				Food waste composting	[311]
Weissella sp. S26/Bacillus sp.ADS3	13.2			Xylose	[312]
*Enterobacter aerogenes* ATCC 29007	46.02	0.41		Mannitol	[313]
*Thermoanaerobacterium aotearoense* LA1002-G40	78.5	0.85	1.63	Mixed bakery waste	[314]
*Lb. sanfranciscensis* MR29	2.85	0.057		Wheat straw biomass	[315]
*Lb. rossiae* GL14	0.96	0.0192			
*Lb. frumenti* H10	1.90	0.038			
*Lb. rossiae* M2	1.54	0.0308			
*Lb. crustorum* W19	2.94	0.058			
*Lb. sanfranciscensis* MW15	4.56	0.0988			
*Lb. helveticus* DSM 20075	2.03	0.0406			
*Lb. delbrueckii subsp. bulgaricus* MI	4.74	0.0948			
*Lb. delbrueckii subsp. bulgaricus* DSM 20081	4.81	0.0962			
*Leuconostoc mesenteroides* NRRL B 512	60.2		1.25	Sugarcane juice	[316]
*B. coagulans* LA1507 and *Lactobacillus rhamnosus* LA-04-1 (Mixed culture)	118		1.84	Sweet sorghum juice	[317]
Engineered *Pediococcus acidilactici*	130.8		1.82	Wheat straw	[318]
*Streptococcus* sp.(indigenous consortium)	50–69		1.27–2.93	Highly viscous food waste	[319]
*Streptococcus* sp.	66.5	0.33	3.38	Mixed food waste	[320]
*Bifidobacterium longum*	0.51			Cheese whey	[321, 322]
***Bacillus* strains**					
***B. coagulans***					
*B. coagulans*	20.1	0.60	0.93	Sucrose	[4]
*B. coagulans* 36D1	80	0 0.80	0.30	Cellulose	[151]
*B. coagulans* strains 36D1	92.0	0.77	0.96	Paper sludge	[20]
*B. coagulans* strains P4–102B	91.7	0.78	0.82	Paper sludge	[20]
*B. coagulans* SIM-7 DSM 14043		0.96	9.9	Glucose	[24]
*B. coagulans* DSM 2314		0.27		Wheat straw	[323]
B*. coagulans* strain 36D1	103.6	0.93	0.71	Glucose	[151]
*B. coagulans* strain 36D1	102.3	0.86	0.71	Xylose	
*B. coagulans* NBRC 12583	2			Sludge hydrolyzate	[324]
*Alkaliphilic Bacillhilic*				Sugars	[13, 14]
*B. coagulans* strain IPE22	46.12			Wheat straw	[33]
*B. coagulans* C106	83.6–215.7		4–7.5	Xylose	[325]
*B. coagulans* NBRC12583				Kitchen refuse	[27]
***Bacillus strains***					

*B. coagulans*	60.7	0.71	2.68	Municipal solid wastes	[112]
*B. coagulans* DSM2314	58.7–70.4	0.83–0.73	1.14–1.81	Sugarcane bagasse	[326]
*B. coagulans*	79.4–93.7			Glucose, xylose and cellobiose	[327]
*B. coagulans* BCS13002	11.75			Gelatinized corn starch	[328]
	0.26			Corn starch	
*B. coagulans*	99.1		1.38	Glucose	[329]
*B. coagulans*	145		1.5	Glucose	[330]
*B. coagulans*	110	0.86	1.29	Cassava bagasse	[304]
*B. coagulans* MA-13	29.7–33.7	0.92		Lignocellulosic hydrolysate	[331]
*B. coagulans* JI12		0.97		Oil palm empty fruit bunch hydrolysate	[332]
*B. coagulans* WCP 10-4	210	0.955	3.5	Glucose or corn starch	[333]
*B. coagulans* C106	83.6	0.983	7.5	Xylose	[334]
*B. coagulans* strainIPE22	38.73	0.813	0.39–0.65	Pretreated wheat straw	[335]
*B. coagulans*		0.94	0.33	Corn stover hydrolysate	[336]
*B. coagulans*	165.7 168.3	0.92 0.88	1.6 2.1	Glucose Glucose/Cane molasses	[337]
*B. coagulans* strain AD		1.4	3.69	Corn stover hydrolysate	[338]
*B. coagulans* strain IPE 22	7.52–56.13	0.13–0.94	0.31–2.77	Single sugar (glucose, xylose, arabinose)	[339]
	49.14–51.47	0.82–0.86	2.05–3.08	Mixed sugar (glucose + xylose + arabinose)	
	50.48–53.51	0.89–0.92	2.97–3.16	Corn cob hydrolysate	
*B. coagulans* L-LA 1507	78–97.5	0.325–0.406	1.25–3.25	Corn stover	[340]
*B. coagulans* AT107	98.8	0.80–0.92	1.25–3.15	Alfalfa green juices and clover green juice	[341]
*B. coagulans*	79.1		0.76	Lignocellulosic corncob residue	[342]
*B. coagulans*	92.5	0.578	2.01	Dilute ethylediamine pre-treated rice straw	[343]
*B. coagulans + B. thermoamylovorans*.	39.2		1.09	Kitchen refuse medium	[118]
*B. coagulans* IPE22	68.72	0.99	1.72	Inedible starchy biomass	[344]
*B. coagulans* LA-15-2	117		2.79	White rice bran	[345]
*B. coagulans* A166	61.1		0.94	Municipal solid waste	[346]
B. subtilis ZM63, B. cereus, *Paenibacillus polymyxa* and *B. cereus*				Glucose + Zn^+2^	[205]
***B. licheniformis***					
*B. licheniformis* TY7	40.0	-	2.50	Kitchen refuse	[27]
*B. licheniformis* TY7	24–40	1.29–1.35		Kitchen refuse	[34, 347]
***B. subtilis***					
*B. subtilis* MUR1 (mutant)	143.2	90.3	2.75	Glucose	[36]
***B*. sp.**					
*B. longum* NCFB 2259		0.51–0.82	0.3–0.7	Cheese whey	[181, 348]
*B.* sp.36D1			0.60	Sugar cane bagasse	[349]
*B.* sp. Na-2	106	0.94	3.53	Glucose	[38]
*B.* sp. WL-S20	225	0.993	1.04	Peanut meal and glucose	[16]
	180	0.98	1.61	Peanut meal and glucose	[16]
*B.* sp. 2-6	107	0.95	2.9	Glucose	[40]
*B.* sp. Na-2	118	0.97	4.37	Glucose	[39]
*B.* sp. P38	180	0.96	2.4	Cellulosic hydrolysate	[37]
***E. coli***					
Engineered *E. coli*	60–62.2	0.80–0.90		Glucose	[348]
Engineered E. coli	45.5–51.8	0.91–0.99		Glucose	[52]
Engineered *E. coli*	40	0.93		Xylose	[57]
Engineered *E. coli*	56.8	0.88	0.94	Glycerol	[350]
*E. coli* AC-521	85	0.85	1.0	Sucrose	[54]
*E. coli* K12 strain	32	0.85	0.44	Glycerol	[59]
*E. coli*	75	0.85	1.18	Molasses	[351]
lactogenic *Escherichia* *coli* strain JU15	40	0.6		Corn stover	[352]
*E. coli* BW25113 (DpflA) (engineered)	5.2	22.5	0.06	cellobiose	[353]
	4.3–5		0.22–0.25		
	5.3	29.6	0.11	Glucose	
***E. coli***					

*E. coli* MG1655-LA02Δdld (engineered)	45	0.83	0.5	Glycerol	[59]
*E. coli* strain CICIM B0013-070 (pUC-ldhA) (engineered)	111.5	0.78	2.80	Glycerol	[354]
Engineered *E. coli*	50	0.90	0.60	Glycerol	[53]
Engineered *E. coli* RR1	62.6			Glucose	[13, 14]
***Corynebacteria glutamicum***					
*C. glutamicum*	120	0.865	~. 4.0	Glucose	[48]
*C. glutamicum*				L-arabinose	[45]
*C. glutamicum*				Xylose	[46]
*C. glutamicum*				Glucose, fructose, sucrose, ribose	[355]
*C. glutamicum*	60.27			D-ribose	[51]
***Achromobacter denitrifleans NBRC* 12669**	3.9	0.41	–	Glycerol	[195]
**Fungi**					
***Rhizopus* sp.**					
***R. oryzae***					
*R. oryzae* ATCC 52311	83.0	0.88	2.6	Glucose	[70]
*R. oryzae*	62	72%	2.5	Glucose	[13, 14]
*R.* sp. MK-96-1196*R.* sp. MK-96-1196	33.3	0.93	1.80	Cull potato glucose	[356]
*R. oryzae*	83	65%	1.6	Glucose	[13, 14]
*R. oryzae*	71.5	71%	-	Glucose	
*R. oryzae*	-	70%	-	Glucose	
*R. oryzae*	40	78%	4.6	Glucose	
*R. oryzae*	-	-	6.2	Glucose	
*R. oryzae*	-	65%	-	Glucose	
*R. oryzae*	112–173	78–94%	2.8–5.6	Glucose	[357]
*R. oryzae*	104.6	87	1.8	Glucose	[13, 14]
*R. oryzae*	60		2.9–6.2	Glucose	[13, 14]
*R. oryzae*	-	-	2.91	Glucose	[72, 77]
*R. oryzae* NRRL 395	104.6	0.87	1.8	Glucose	[153]
*R. oryzae* NRRL 395		0.87–0.90	1.8–2.5	Glucose	[86]
*R. oryzae* R1021		0.77		Glucose	[83]
*R. oryzae* NRRL 395		≈1	1.65	Corn	[86]
*R. oryzae* RBU2-10			1.84	Rice	[358]
*R. arrhizus* DAR 36017			1.3–1.6	Potato	[172]
*R. ory*zae HZS6		0.80	0.99	Corncob	[155]
*R. oryzae* NRRL395			0.31	Corncob	[65]
*R.* sp. MK-96-1196	24.0		0.3	Corncob	[63]
*R. oryzae* NRRL 395	49.1		0.7	Waste paper	[153]
*R. oryzae* GY18	115	0.81	1.6	Glucose	[359]
*R. oryzae* GY18	80.1	0.89	1.67	Sucrose	[359]
*R. oryzae* GY18	68.5	0.85	0.57	Xylose	[359]
*R. oryzae* NBRC 5378	14.4	–	0.56	Xylose	[69]
R. oryzae ATCC 9363	113	0.90	4.3	Glucose	[360]
*R. oryzae* NRRL 395	91.0	0.76	2.02	Corn starch	[13, 14]
*R. oryzae*	103.7	–	2.16	Glucose	[84]
	81–95	–	3.4–3.85	Glucose	[84]
*R. oryzae* NBRC 5384	145	0.95	1.42	Glucose	[361]
	231	0.93	1.83	Glucose	[361]
*R. oryzae*	51.7	0.68		Oat	[362]
*R. oryzae*	173.5	0.86	1.45	Tobacco waste water-extract and glucose	[363]
*R. oryzae* As3.819	80.2			Glucose	[364]
*R. oryzae*	463.18	0.83	2.76	Cassava pulp	[365]
*R. oryzae*	75.28	0.5	1.05	Cassava pulp hydrolysates	[366]
*R. arrhizus*	68.8	0.93	0.72	Honeycomb matrix	[367]
*R. arrhizus*	75.1	0.63	1.54	Glucose	[368]
*R. arrhizus*	1.2			Pretreated dairy manure	[369]
*R. arrhizus*	34–60.3	0.34–0.60		Xylo-oligosaccharides manufacturing	[370]
*R. arrhizus* UMIP 4.77	10	0.26	0.27	Wheat straw	[371]
***Rhizopus sp.***					
*R. arrhizus*	46.78		0.97	Animal feeds from Sophora flavescens residues	[372]
*R. microsporus*	84.3–119	0.84–0.93	1.25	Liquefied cassava starch	[373]
*R. arrhizus*	103.8			Waste potato starch	[374]
*Monascus ruber*	129–190	0.58–0.72	0.91–1.15	Glucose	[375]
Engineered *Aspergillus brasiliensis* from *Rhizopus oryzae*	13.1–32.2		0.26–0.47	Glucose	[376]
*Aspergillus niger*	7.7		0.13	Glucose	[377]
**Yeast**					
Engineered *P. stipitis*: LDH from *L. helveticus* (integrated, 1 copy)	15–58	0.58	0.6	Glucose	[100]
***Saccharomyces***					
Engineered *S. cerevisiae* LDH from *L. casei* (multicopy vector)	12 g/L			Glucose	[13, 14]
Engineered *S. cerevisiae* LDH from *L. casei*	8.6	0.04		Glucose	[13, 14]
Engineered *S. cerevisiae* LDH from *B. taurus* (integrated, 1 copy)	20			Glucose	[13, 14]
Engineered *S. cerevisiae* LDH from *B. taurus* (multicopy plasmid)	11.4			Glucose	[13, 14]
Recombinant Saccharomyces cerevisiae CENPK2	2.22			Food waste biomass	[378]
Engineered *S. cerevisiae* OC-2T T165R	~45–50		~0.45–1.6	Glucose	[379]
Engineered *S. cerevisiae* LDH from *B. taurus* (multicopy plasmid)	6.1			Glucose	[13, 14]
Engineered *S. cerevisiae* LDH from *L. plantarum* (integrated, 1 copy)	58			Glucose	[380]
Engineered *S. cerevisiae* LDH from *L. casei* (integrated, 2 copy)	1.6 mol/96h			Glucose	[92]
Engineered *S. cerevisiae* LDH from *B. taurus* (integrated, 2 copies)	50.6			Glucose	[381]
Engineered *S. cerevisiae* LDH from *B. taurus* (integrated, 6 copies)	120			Glucose	[381, 382]
Engineered *S. cerevisiae* LDH from *L. mesenterioides* (D-LDH, integrated,					
2 copies)	53.2			Glucose	[383]
Engineered *S. cerevisiae* LDH from *B. taurus* (integrated, 2 copies)	82.3			Glucose	[95]
Engineered *S. cerevisiae* HDH from *R. oryzae* (multicopy plasmid)	38			Glucose	[96]
Engineered *S. cerevisiae* HDH from *L. plantarum* (multicopy plasmid)	70	0.93		Glucose	[98]
Engineered *S. cerevisiae* LDH from *B. taurus* (integrated, 8 copies)	80			Glucose	[97]
Engineered *S. cerevisiae* LDH from *B. taurus* (integrated, 2 copies)	74.1			Glucose	[97]
Engineered *S. cerevisiae* LDH from *B. taurus* (integrated, 2 copies)	71.8			Glucose	[97]
Engineered *S. cerevisiae*	122	0.61		Cane juice	[67]
*S. cerevisiae*	117	0.58		Glucose	[384]
Recombinant Saccharomyces cerevisiae	60.3	0.646	2.8		[385]
Engineered *Issatchenkia orientalis*: LDH from *L. helveticus* (integrated, 1 copy)	66			Glucose	[386]
Engineered *Issatchenkia orientalis*: LDH from *L. helveticus* (integrated, 1 copy)	70			Glucose	[387]
**Candida**					
**Candida utilis**					
Engineered *Candida utilis*: LDH from	93.9	0.91	2.18	Xylose	[388]
Engineered *Candida utilis*: LDH from *B. taurus* – optimised (integrated, 2 copies)	103.3				[104]
**Candida *boidinii***					
Engineered *Candida boidinii*: LDH from *B. taurus* – optimized (integrated, 1 copy)	85.9			Glucose	[99]
***Candida sonorensis***					
*Candida sonorensis*	92	0.94	4.9	Glucose	[100]
*Candida sonorensis*	40	0.60		Glucose	[389]
Engineered Candida glycerinogenes from Rhizopus oryzae				Glucose	[390]
**Kluyveromyces**					
*K. marxianus*	8.8	0.24	4.3		[219]
Engineered *K. marxianus from actobacillus plantarum*	122–130			Jerusalem artichoke tuber powder	[391]
Engineered *K. marxianus from Homo sapiens* (*Hs*LDH), *Bacillus subtilis* (*Bs*LDH), *Bacillus megaterium* (*Bm*LDH), *Lactococcus lactis* (*Ll*LDH), *Rhizopus oryzae* (*Ro*LDH), and *Plasmodium falciparum* (*Pf*LDH)	25–105			Alkali-pretreated corncob	[392]
Engineered *K.* marxianus *L*DH from *L. helveticus* (integrated into PDC1 locus)	99			Glucose	[106]
Engineered *K.* marxianus *L*DH from *L. helveticus* (integrated into PDC1 locus)	9.1			Glucose	[106]
Engineered *K. lactis L*DH from *B. taurus* (low copy number plasmid, 5 copies)	109	0.91		Glucose	[13, 14]
Engineered *K. lactis L*DH from *B. taurus*(multicopy plasmid)	60	0.85		Glucose	[93]
Engineered *K. lactis L*DH from *B. taurus*		0.58–1.00		Glucose	[93]
**Schizosaccharomyces**					
Engineered *Schizosaccharomyces pombeL*DH from *R. oryzae*	80–100			Glucose	[393]
*Schizosaccharomyces pombe*	24.4		0.45	Cellobiose	[394]
*Schizosaccharomyces pombe*	60.3		0.45	Glucose	[395]
*Schizosaccharomyces pombe*	112		2.2	Glucose	[396]
**Microalgae and cyanobacteria**					
Engineering *Synechocysti*s sp. PCC 6803	3.31			Glucose	[397]
Engineering of Schizosaccharomyces pombe	24.4–25.2	0.68–0.81		Glucose and cellobiose	[394]
**Consortia**					
MAR compost	34.2	0.54		Kitchen refuse	[113]
waste activated sludge (*Bacillus, Clostridiaceae, Lactobacillus* and *Peptostreptococcaceae)*	26.63–29.77			Food waste	[398]
Naturally inhabiting bacteria in garbage	64	0.62		Kitchen refuse	[114]
Naturally inhabiting bacteria in garbage	37.7	0.58		Garbage	[399]
Anaerobic digestion sludge	4.17	0.429		Glucose	[400]
Anaerobic digestion sludge	23	0.92		Glucose	[401]
Excess sludge	8.5	1.06		Sucrose	[402]
Naturally inhabiting bacteria in garbage	˂27.5			Kitchen refuse	[298]
Microbial consortium CEE-DL15 *Clostridium sensustricto* (57.29%), *Escherichia* (34.22%), and *Enterococcus* (5.32%)	112.3 18.5	0.81	4.49	Sugarcane molasses	[403]
Anaerobic activated sludge as inocula	28.4	0.46		Methanogenic sludge and fresh food waste	[404]

Cases with no data indicate absence of results in the cited reference.

LAB can metabolize glucose into LA, acetic acid (AA), formate, ethanol, diacetyl, acetoin, and carbon dioxide (CO_2_ gas detection is a diagnostic test for heterofermentative from homofermentative fermentation) [14]. The heterofermentative LAB can use the phosphogluconate pathway (with a theoretical yield of 0.5 g/g) and phosphoketolase pathway (with a theoretical yield of 0.6 g/g), when metabolizing hexose and pentose sugars, respectively [13, 14].

The utilization of heterofermentative LAB as dairy starter cultures are not common due to CO_2_ release and simultaneous production of LA and other organic acids, considered as defects which induce several problems in the products, including bloated packaging and cracks in dairy products and hard cheeses, respectively. Heterofermentative LAB includes mainly *Oenococcus*, *Leuconostoc*, and some *Lactobacillus* spp., and the main heterofermentative *Lactobacillus* spp. are *Lb. brevis*, *Lb. fermentum*, and *Lb. reuteri.*

#### 2.1.2 *Bacillus* strains

*Bacillus* also has metabolic capacity to produce LA. There are several advantages to the use of *Bacillus* spp. relatively to the LAB. The use of *Bacillus* spp., allows reducing the LA production cost, because: (1) they can grow and ferment in mineral salt media with inexpensive nitrogen sources such as steep corn liquor or (NH_4_)_2_SO_4_, temperature (50–55 °C) and pH (6–6.5); (2) media sterilization before the fermentation process can be avoided due to the high temperature of LA fermentation process (>50 °C) and so do not need also cooling after medium sterilization, with considerable costs reduction; (3) they can utilize all sugars from lignocellulose biomasses, due to the ability to metabolize pentose sugars via the pentose phosphate pathway and hexose sugars via the EMP pathway; (4) all strains of *Bacillus* produce only L-LA [15]; (5) they can convert substrates to LA with high yield or high productivity; (6) some strains namely *B. coagulans* JI12 was tolerant to both furfural (4 g/l) and acetate (20 g/l). Neither pre-detoxification nor separation of fermentable sugars from lignin was needed before the fermentation. Meng et al. [16] and Patel et al. [17] reported that the alkaliphilic *Bacillus* sp. WL-S20 and *B. coagulans* 36D1 produced L-LA at concentration and yield of (225 g/L and 0.993 g/g) and (92.0 and 0.96 g/g), respectively. *Alkaliphilic Bacillus* sp. WL-S20 generated L-lactic acid in fed-batch fermentation at pH 9.0, which would reduce a risk of the contamination during fermentation and also can produce lactic acid in thermal fermentation (≥50 °C) [16]. *Bacillus* spp. have been accredited by European Food Safety Authority (EFSA) and Food and Drug Administration (FDA) to the Qualified Presumption of Safety (QPS) list and Generally Recognized As Safe (GRAS) status for applications in livestock production [18]. Some *Bacillus* strains could produce LA, including *B. coagulans* [19, 20, 21, 22, 23, 24, 25, 26, 27, 28, 29, 30, 31, 32, 33]*, B. stearothermophilus* [13, 14], *B. licheniformis* [34] thermophilic *B. licheniformis* [35], *B. subtilis* [36], *Bacillus* sp [37, 38, 39, 40]. and alkaliphilic bacilli such as *B. circulans* var. *alkalophilus* ATCC 21783, *B. alcalophilus* sp. *halodurans* ATCC 27557, *B. alcalophilus* ATCC 27647, alkaliphilic *B.* sp. WL-S20 and *B.* sp. 17-1 ATCC 31007 [16].

#### 2.1.3 Corynebacterium glutamicum

*Corynebacterium glutamicum* is an aerobic Gram-positive bacterium that has been reported to be able to excrete amino acids (L-lysine and L-glutamate) and also small amounts of mix-organic acids (LA, succinic acid (SA), and AA) in industrial production. The organic acids production reported has occurred under oxygen deprivation conditions (anaerobic condition) due to cell growth inhibition and acceleration of mix-organic acids production from various sugars, including D-glucose [41, 42, 43]; L-arabinose [44]; D-glucose and L-arabinose [45] D-xylose and D-glucose [46] and D-xylose, D-cellobiose and D-glucose [44] in mineral salts medium [13]; *C. glutamicum* is engineered and has highly potential bacterium that can produce LA with high yield and productivity without requiring complex nutritional compounds. *C. vitaeruminis* MTCC 5488 produced 38.5 g/l LA in fed-batch fermentation [13]. Meanwhile, *C. glutamicum*, as well as *E. coli (section 1-4)*, have extremely low tolerance to acidic condition; hence LA production needs to be performed at pH-values about 7.0.

However, the simultaneous production of LA and the formation of several organic acids such as SA and AA resulted in a low LA production yield which should be improved [47]. Several types of research strategies were attempted to increase the LA production by *C. glutamicum* fermentation, through the promotion of medium conditions changes or by using engineering methodologies, such as:

A) Inui et al. [41] and Okino et al. [48] reported a novel system which consists in a reactor containing high-density cells (HDC) of *C. glutamicum* (the cell concentrations were almost 10-fold higher than those commonly used for batch fermentation) that could lead to the high volumetric productivity of LA. According to the results of Yukawa et al. [49], LA was produced by using the *C. glutamicum* R strain under an HDC condition.
B) Manipulation of *C. glutamicum* could produce D-lactic acid at higher productivity and purity compared with the parental strain. Simultaneously knock out of the L-LDH gene, and over expression of the D-LDH encoding gene was performed by inserting this gene into *C. glutamicum* from *Lb. delbrueckii* [43] and *Lb. Bulgaricus* [42]*.*

Song et al. [50] reported an engineered *C. glutamicum* strain that can produce D-lactyl-CoA (by D-LDH and propionyl-CoA transferase) and 3-hydroxybutyryl-CoA (by β-ketothiolase and NADPH-dependent acetoacetyl-CoA reductase) from glucose, under several enzymatic reactions. Copolymerization of 3-hydroxybutyryl-CoA and D-lactyly-CoA by using lactate polymerizing enzyme reaction resulted in the production of poly (LA-co-3HB) with high LA fractions (96.8 mol%) [50].

C) On the other hand, some studies reported that an engineered *C. glutamicum* could utilize pentose sugars including xylose [46] and arabinose [45], as well as hexose sugars, such as galactose and glucose. Kawaguchi et al. [46] inserted the genes xylA and xylB from *E. coli* into the *C. glutamicum* R strain that encodes xylose isomerase and xylulokinase, respectively, using a multicopy plasmid under the controlled promoter condition. Both the expression of xylA and xylB genes with xylose utilization ability could enhance the growth rate and production pattern of organic acid including L-LA and SA with interesting productivities (29 and 17 mmol/l/h) and yields (0.53 and of 0.25 g/g), respectively [46]. Kawaguchi et al. [45] performed another study in order to gain arabinose utilization ability, throughout the expression of genes araA, araB and araD (encoding arabinose isomerase, ribulokinase, and ribulose-5-phosphate 4-epimerase, respectively) from *E. coli* into the *C. glutamicum* R strain. The results showed that the engineered *C. glutamicum* could consume arabinose, through successful arabinose genes expression, leading to the production of L-LA (3.4 mmol/h/g dry cell), SA and AA. This L-LA was produced using a mixture of sugars (arabinose and glucose), being the glucose consumption rate (0.76 g/h/g dry cell) significantly higher than the arabinose counterpart (0.06 g/h/g dry cell) [45].
D) Pyruvate kinase (Pyk) plays a key role in the production of pyruvate and ATP in glycolysis pathway and, moreover, as an essential factor in controlling the carbon flux distribution. *C. glutamicum* only contains one Pyk (pyk1NCgl2008). Moreover, recently Chai et al. [51] found NCgl2809 as another novel pyruvate kinase (Pyk2) in *C. glutamicum.* These authors grew an engineered *C. glutamicum* containing Pyk1 or Pyk2 on D-ribose conditions, being the LA production enhanced by overexpression of either Pyk1 or Pyk2, due to the increase of the activity of the Pyk enzyme. They found that fermentation by the overexpression of Pyk2 in WTΔpyk1 *C. glutamicum* strain could increase LA production to 60.27 ± 1.40 g/L (about 47% higher than the parent strain) under oxygen deprivation condition.

#### 2.1.4 Escherichia coli

Wild-type *E. coli* is capable of growing and producing LA using hexoses and pentoses sugars fermentation with production of a mixture of organic acids (AA, SA, and formic acid (FA)) and ethanol [47, 52]. Moreover, they can grow on broth with more straightforward nutrient requirements compared to the conventional LAB.

Engineered *E. coli* showed improved LA fermentation efficiency compared with wild *E. coli* [13, 14, 52]. These engineered strains were manipulated by (1) replacement of D-LDH with L-LDH from LAB, bovine and other sources [13, 14, 52].; (2) prevention synthesis of racemic mixtures of D- and L-lactates by omission of methylglyoxal bypass route and consequently its accumulation; (3) avoiding of the undesired utilization of L-lactate by blocking the aerobic L-LDH [53]. Engineered *E. coli* strains can grow and produce LA from several disaccharides including sucrose [54, 55] and monosaccharides (hexoses and pentoses) including glucose [13, 14, 52, 56, 57, 58], xylose [56], and also glycerol [13, 14, 59, 60]. Some researchers reported that engineered *E. coli* strains produce D-LA by the homofermentative substrate pathway that causes over-expressing of LA. However, engineered *E. coli* strains had shown several disadvantages, such as low productivity (≤1.04 g/L/h) and low tolerance to low pH conditions due to LA production, in comparison with LAB [13, 14, 57].

### 2.2 Filamentous fungi

Filamentous fungi are another microbial source that can produce LA. Numerous species of the genus *Rhizopus* such as *R. oryzae* and *R. arrhizus* can produce L-LA (as the main product) fumaric acid, and ethanol from different carbon sources [64]. Among carbon sources, they aerobically metabolize glucose to produce LA. However, there are several renewable carbon resources for LA production by *Rhizopus* strains, which include corncob hydrolysate [61, 62, 63, 64, 65]; xylose [66, 67], glucose [13, 14, 68], wheat straw [69], paper pulp sulfite liquor [70], chicken feather protein hydrolysate [71], molasses [71], cassava pulp hydrolysis [72], potato hydrolysate [73], and glycerol enriched with lucerne green juice and inorganic nutrients [74]. Media containing nitrogen sources lead to a fast growth that induces the production of chitin instead of LA [15]. On the other hand, lack of a nitrogen source leads to a decreased cell activity and product formation in long-term cultivation [15]. Two solutions to overcome this drawback was: 1) cells morphology affected LA productivity and yield (for example, fungal pellets instead of spores [73]; 2) medium composition manipulation by using low nitrogen sources and high content of carbon sources could enhance LA production [73]. Urea is one of the nitrogen sources used by genus *Rhizopus* that when added periodically within the production phase can avoid biofilm overgrowth, postpone sporulation, and retain high cell viability and LA productivity [72].

There are some advantages and disadvantages of using *Rhizopus* strains for LA production. Some benefits of *Rhizopus* strains in comparison to LAB include: 1) their amylolytic properties (containing amylolytic enzyme activity) that can convert various starchy biomasses directly to L-LA without prior saccharification process [75]; 2) simple medium requirements [76-78]; 3) their filamentous or pellet growth in fermentation medium facilitate their separation from fermentation broth, which can lead to lower-cost downstream process [79]; 4) fungal biomass is a worth fermentation by-product. On the other hand, *R. oryzae* is an obligate aerobe and requires vigorous aeration, usually above an oxygen transfer rate of 0.3 g O_2_/L/h [80, 81]. A disadvantage of using fungi is related with the different morphology of growth under fermentation, which includes extended filamentous appearances, pellets, mycelial mats, and clumps that significantly affect LA productivity and rheology of broth medium. Their morphology can affect the oxygen supply and mass transfer. In fungal fermentation, the low LA productivity (below 3 g/(L·h)) is a result of the low O_2_ mass transfer and synthetic route shift toward production of other by-products such as ethanol and fumaric acid. The preferable fungal morphology for industrial fermentations is small pellets by several reasons: 1) improved rheology of broth fermentation; 2) enhanced mass transfer in fermentation broth; 3) can be continuously utilized by using repeated batch fermentation for long operations [82].

Some researchers investigated fungi morphology that enhances the LA productivity. Abdel-Rahman et al. [13, 14] verified that high LA production was obtained by cotton-like mycelial flocs morphology, which was formed by the culture of *R. oryzae* in the air-lift bioreactor*.*

Several reports attempted to achieve high yield and productivity of pure L-LA with higher cell density by fungal fermentation [71, 83, 84], including the following:

1. Immobilization techniques, being *Rhizopus oryzae* immobilized for L-LA production [13, 14, 85, 86], but entrapment of fungal cells on matrixes revealed to be time-consuming.
2. Controlling the production of undesirable by-products, mainly ethanol and fumaric acid leads to higher LA productivity [87, 88, 89].
  2.1. Addition of alcohol dehydrogenase (ADH) inhibitor into the fermentation medium (i.e., 1,2-diazole and 2,2,2-trifluoroethanol) as an active inhibitor to decrease ethanol production and lactate dehydrogenase (LDH), as a useful promoter to increase LA and cell biomass production [90].
  2.2. Metabolic engineering of the strain by deleting the alcohol dehydrogenase and malate dehydrogenase genes, thus shifting the metabolic flux, increasing LA production and yield [89].

As far as we are aware, there are no reports that include other fungi to produce LA. The fungus, *Aspergillus niger* together with *Lactobacillus* sp. was used for LA production. The strategy, in this case, was that fungi enzymes would perform saccharification and de-polymerization of carbohydrate polymers to produce fermentable sugars to be used by the bacterium [10, 91].

### 2.3 Yeasts

Presently, LAB is the main microorganisms used to LA production. However, there is one problem associated to their use; their low pH sensitivity leads to the use of large amounts of neutralizing agents, including CaCO_3_ and results in the production of gypsum in fermentation medium [92]. Comparatively, yeasts versus bacteria, yeasts can tolerate low pH which leads to a reduction for the need of neutralizing agents and downstream processing cost. The worst important disadvantage of using wild-type yeasts is the reduced LA production as the main product. Nevertheless, engineered yeasts are the best solution to overcome this drawback.

Engineering yeast manipulation has been studied to obtain high LA productivity and yield, due to cancelation of pyruvate decarboxylase and/or pyruvate dehydrogenase activities, which results in the partial or full substitution of ethanol by LA production [93]. In order to improve the natural acid resistance of yeasts, lactic acid productivity has been enhanced by inserting the gene encoding L(+)-LDH from heterologous sources. The bovine gene encoding LDH has been successfully expressed in both *Candida utilis* and *Saccharomyces cerevisiae*, and the gene encoding LDH from *Lb. helveticus* has been expressed in *Candida sonorensis* [1]. Different research teams have been attempting to produce lactate from engineered yeasts genera including *Saccharomyces cerevisiae* [13, 14, 92, 94, 95, 96, 97, 98], *Candida* spp. [99], *Kluyveromyces lactis* [13, 14, 93], *Torulaspora delbrueckii* [13, 14], *Pichia stipites* [100] and Z*ygosaccharomyces bailii* [101].

#### 2.3.1 *Saccharomyces cerevisiae*

*Saccharomyces cerevisiae* is one of the more permissive organisms used for LA production due to a high intrinsic tolerance to low pH-values. This characteristic should give to *S. cerevisiae* several advantages over LAB and *Bacillus* spp. Firstly, it is a microorganism resistant to low pH and can grow aerobically on glucose sources with the basic anaerobic growth factors including oleic acid, nicotinic acid, and ergosterol.

Engineered *S. cerevisiae* can efficiently produce d-lactic acid due to its capability to grow fast under anaerobic and aerobic conditions. In transgenic strains, the coding section of pyruvate decarboxylase 1 (*PDC1*) was completely eliminated, and one or several copies of the d-lactate dehydrogenase (d-*LDH*) gene resources were inserted into the genome from mammalian LAB such as *Leuconostoc mesenteroides* subsp. *mesenteroides* strain NBRC3426. This study was for the first time performed by Porro et al., 1995, having achieved an LA production of 20 g/l and productivity up to 11 g/L/h using engineered *S. Cerevisiae* [13, 14].

#### 2.3.2 Candida

##### 2.3.2.1 Candida sonorensis

*Candida sonorensis* as a methylotrophic yeast that can ferment hexose (i.e., glucose) and pentose sugars (i.e., xylose and arabinose) to ethanol. They tolerate acid environments and require simple growth medium. *C. sonorensis* was manipulated by insertion of L-LDH genes from *Lb. helveticus*, *B. megaterium*, and *R. oryzae*. Multiple LDH gene copies were expressed to produce suitable mutants for LA production, which produced LA and ethanol. In order to increase the LA productivity, ethanol production was stopped by the elimination of two pyruvate decarboxylase genes (PDC) 1 and 2, being these the primary enzymes contributing to ethanol production. This modification (*C. sonorensis* expressing *L. helveticus* LDH) did not affect cell growth and resulted in the accumulation of lactate up to 92 g/l with a yield of 0.94 g/g glucose without ethanol production [102]. In another work, engineered *C. sonorensis* (L-lactic acid dehydrogenase (ldhL) from *Lb. helveticus*) was reported to produce 31 g/l LA from 50 g/l D-xylose free of ethanol [103].

##### 2.3.2.2 Candida boidinii

Genetic engineering can be used to construct a crabtree-negative methylotrophic haploid of *Candida boidinii* that can efficiently produce high amounts of L-LA [99]. The ethanol production of *C. boidinii* was 17% reduced by knocking out of the PDC1 gene encoding pyruvate decarboxylase when compared with the wild-type strain and with simultaneous heterologous expression of the bovine L-LDH gene resulted in 85.9 g/l of LA with a productivity of 1.79 g/l/h [99].

##### 2.3.2.3 Candida utilise

*Candida utilis* as crabtree-negative yeast is currently used for the production of several valuable chemicals, including glutathione, single cell protein, and RNA. The most pertinent advantage of *C. utilis*e for LA production is the use of inexpensive substrates for growing, which includes pulping-waste liquors. In the study performed by Ikushima et al. [104], an engineered *C. utilis*e strain produced L-LA with high efficiency. These authors reduce ethanol production (as a by-product of L-LA) by knocking out the gene encoding pyruvate decarboxylase (CuPDC1), and then two copies of the bovine L-lactate dehydrogenase (L-LDH) gene were inserted into the CuPdc1-null strain genome. The engineered *C. utilis*e produced 103.3 g/l of L-LA with 95.1% conversion of basal medium and 99.9% purity.

#### 2.3.3 Kluyveromyces

##### 2.3.3.1 Kluyveromyces lactis

*Kluyveromyces lactis* is crabtree-negative yeast which was used for LA production after genetic modification. In comparison with some other yeasts strains, such as *S. cerevisiae*, which have a pyruvate decarboxylase (PDC) with two active structural genes (PDC1 and PDC5) [93], *Kluyveromyces lactis* has expressed PDC activity with a single gene, *KlPDC1*. The omission of *KlPDC1* leads to production strains without PDC activity and increase LA production with free ethanol. The intense competition for pyruvate consumption by homologous PDC and heterologous LDH activities leads to a low LA yield, due to the simultaneous production of ethanol and LA. On the other hand, the elimination of pyruvate decarboxylase gene (KlPDC1), as a single gene with PDC activity in *K. lactis,* resulted in no ethanol production [93]. In this yeast, the bovine L-lactate dehydrogenase gene (LDH) insertion and decarboxylase gene deletion were sufficient to increase the LA production to 109 g.l^−1^, with a productivity of 0.91 g.l^−1^. h^−1^, and yield 1.19 mol.mol^−1^ of consumed glucose [13, 14]. In another study, the KlPDC1 and pyruvate dehydrogenase (PDH) genes were deleted, being the LDH gene inserted into a wild-type of *K. lactis.* The LA production improved by shifting of pyruvate flux toward homolactic fermentation with a yield level of 0.85 g g^−1^ (being the maximum theoretical yield 1 g.g^−1^) [93].

##### 2.3.3.2 Kluyveromyces marxianus

*Kluyveromyces marxianus* has several advantages which make it economically attractive for commercial-industrial applications, including 1) proliferation occurs at high temperatures (up to 52 °C), reducing contamination control in commercial cultivation, whereas most organisms in an industrial environment cannot be cultivated well at this temperature [105]; 2) *K. marxianus* in enriched media conditions, can grow rapidly with doubling times of 0.75–1 h (37 °C) [105]; 3) Many *K. marxianus* strains can utilize various inexpensive carbon sources and require few additional nutrients [105]. In this yeast, the LA concentration was improved by the insertion of the LDH gene from *B. megaterium* [105]. Also, Hause et al. [106] transformed *K. marxianus* by insertion of the LDH gene (from *Lb. helveticus* and integrated into PDC1 locus) and verified an L-LA production at 9.1 g/L.

#### 2.3.4 Zygosacchromyces

*Zygosaccharomyces bailii* has been suggested as another host for LA [107], due to its ability to tolerate environmental restrictions, including high sugar concentrations, acidic conditions, relatively high temperatures (higher than fermentation process) and LA production levels compared with *S. cerevisiae*. *Z. bailii* has a high growth rate and biomass yield which could improve the fermentation processes of LA production. An engineered *Z. bailii* was produced by heterologous LDH gene expression (from the bacterial L-LDH) to induce the shift of the glycolytic flux towards the lactate production [101, 107] to improve LA production efficiency.

#### 2.3.5 Pichia stipitis

*Pichia stipitis* can utilize pentose and hexose sugars from lignocellulose hydrolysates as substrates to produce ethanol. The deletion of alcohol dehydrogenase 1 (ADH 1) and insertion of L-LDH (from heterologous *Lb. helveticus*) under the ADH1 promoter, led to an engineering *P. stipitis* producing 58 and 41 g/l of LA from 100 of xylose and 94 g/l glucose, respectively. Moreover, ethanol production was reduced by 15–30 % and 70–80 % compared with the wild-type strain, by xylose and glucose utilization, respectively [100].

### 2.4 Microalgae and cyanobacteria

Algae and cyanobacteria are included in the category of photosynthetic microorganisms, and they can grow almost anywhere, with a short harvesting cycle of about 1–10 days and produce various chemicals (including biofuels (H_2_), ethanol, lactic, AA and FA). Algal biomass can be proposed as an alternative candidate to LA production without carbohydrate feed medium costs, being induced in high content of carbohydrates and proteins and also lack lignin [15, 108].

A few reports have evaluated the content of LA production by microalgal species, such as:

1. *Scenedesmus obliquus* strain D3 could produce d-LA as the main fermentation product [13, 14];
2. *Nannochlorum* sp. 26A4 produced LA at 26 g/L with a yield of 70% and optical purity of 99.8% from starch (40% content per dry weight) under dark and anaerobic conditions [109];
3. Biomass of *Nannochloropsis salina* contains 40% lipids, 20% carbohydrates, and 40% proteins. The neutralized and concentrated lipid-free residue has 64.3% of sugars (glucose and xylose). Co-fermentation of *N. salina* and *L. pentosus* under anaerobic fermentation could yield 10.1 g/l of LA with 92.8% of the conversion [110].
4. *Synechococcus elongates* PCC7942 engineered with simultaneous genes expression encoding glucose; lactate and fructose-facilitated diffusion transporter; L-LDH (from *E. coli*) and invertase could produce 600 μM of LA. Similarly, engineered *Synechocystis* sp. PCC6803 by insertion L-LDH gene (derived from *B. subtilis*) could produce of 3.2 mM LA [111].

## 3 Substrates for lactic acid production

### 3.1 Food waste

Food waste can include any compounds from the food production process to the wastes formed by the final consumer. Food waste contain a high amount of carbohydrate which causing it suitable as a substrate for lactic acid fermentation. Regarding to Table 1, numerous studies stated food waste are suitable for lactic acid production such as kitchen residues/refuse and municipal solid wastes [112], model kitchen refuse medium contain water, vegetables, meat/fish and cereals [113], mixes of cooked rice, vegetables, meat, and bean curd [113, 114]; rice, noodles, meat, and vegetables [115, 116]; vegetables such as carrot peel, cabbage, and potato peel, fruit such as banana peel, apple peel, and orange peel, baked fish, rice, and used tea leaves [117, 118]; rice, noodles, meat and vegetables, and unsold bakery products including cakes, breads and pastries [119]; rice, vegetables, and meat [120]; coffee mucilage [119]; and coffee pulp [121].

### 3.2 Carbohydrates

#### 3.2.1 Starchy biomass and sugar plant wastes (malt, molasses and sugar beet juice)

**Figure 2:**
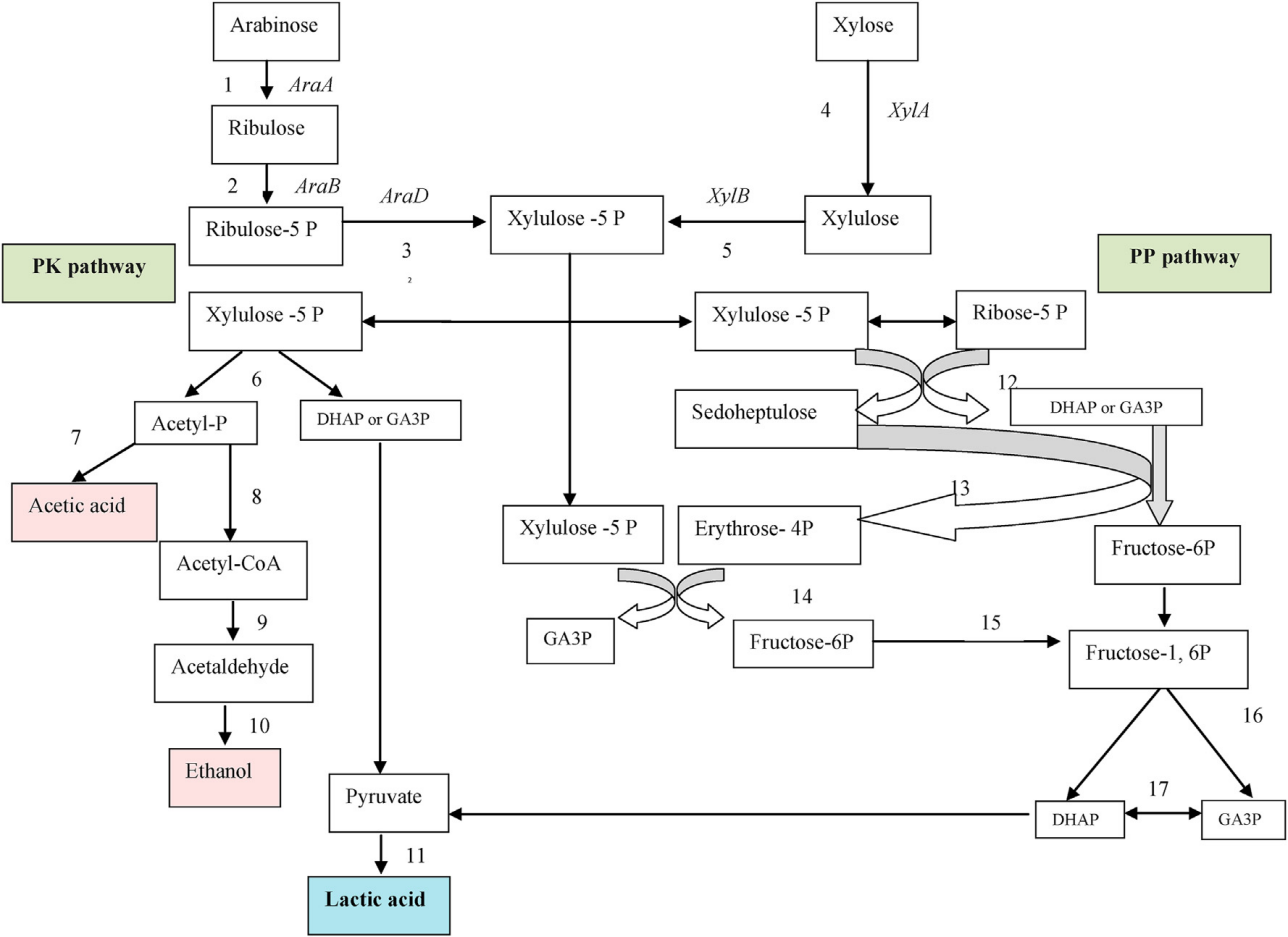
Pathways of lactic acid production from pentose sugars obtained from lignocellulose hydrolysate. Genes *AraA*, AraB, and *AraD* encoding arabinose isomerase, ribulokinase, and ribulose-5-phosphate 4-epimerase, respectively. XylA, and xylB encodes xylose isomerase, and xylulokinase. (1) arabinose isomerase; (2) ribulokinase; (3) ribulose-5-phosphate 3-epimerase; (4) xylose isomerase; (5) xylulokinase; (6) phosphoketolase; (7) acetate kinase; (8) phosphotransacetylase; (9) aldehyde dehydrogenase; (10) alcohol dehydrogenase; (11) lactate dehydrogenase; (12) transketolase; (13) transaldolase; (14) 6-phosphofructokinase; (15) fructose-bisphosphate aldolase; and (16) triosephosphate isomerase. PK pathway and PP pathway are phosphoketolase and pentose phosphate pathway. GA3P: glyceraldehyde-3-P, DHAP: Dihydroxyacetone-P.

Lactic acid can be produced from sugar plant wastes (molasses and sugar beet juice), starchy, and lignocellulosic biomasses (Figure 2).

Disaccharides (lactose and sucrose) and monosaccharides hexoses (glucose, fructose, and galactose) and pentoses (xylose and arabinose) sugars can be fermented by LAB via EMP and/or the pentose PK pathway [122]. Molasses are waste products containing a large amount of sucrose and other essential nutrients, which can derive from sugar cane and sugar beet from sugar manufacturing plants. Several microorganisms can use molasses as a substrate including *Lb. delbrueckii subsp. delbrueckii* mutant Uc-3 [123], *Lb. delbrueckii* NCIM 2025 [124]; *Lb. delbrueckii* NCIMB 8130 [125]; *Lb. delbrueckii* C.E.C.T. 286 [13,14], *Lb. delbrueckii* IFO 3202 [13,14], *Lb. delbrueckii* [126], *Lb. plantarum* [127], *Sporolactobacillus cellulosolvens* [13, 14], *Rhizopus arrhizus* [128], *Lb. casei* M-15 [129], *Bacillus* sp. XZL9 [29] and *E. faecalis* [130]. Shukla et al. (2004) also reported that recombinant *E. coli* strain could produce D-lactic acid from molasses [131]. Raw sugar beet juice with a Brix of at least 60 was used for LA production by lactic acid-producing microorganisms including bacteria (lactobacilli and moderately thermophilic bacilli due to fermentation at relatively high temperature such as *B. coagulans, B. thermoamylovorans*, *Geobacillus stearothermophylus* and *B. smithii,* yeasts and fungi, such as, *Rhizopus* and *Aspergillus* [132]. Malt and date juice are another source for LA production by *Lb. casei* subsp. *rhamnosus* in batch and fed-batch cultures with a maximum LA production level of 89.2 g/L already achieved [133, 134].

There is a great interest to introduce cellulosic and starchy materials as substrates for LA production due to their abundance, low price and for being derived from renewable sources [135]. Amylolytic lactic acid bacteria (ALAB) such as *Lb. plantarum*, *Lb. fermentum* and *Lb. manihotivorans*, *Lb. amylophilus* and *Lb. amylovorus* can ferment starchy biomass into LA due to their α-amylases activity [13, 14, 136, 137]. Some ALAB were isolated from various amylaceous compounds, which include maize and maize-based fermented products [13, 14, 138], potato [13, 14, 73, 138, 139], cassava and cassava-based fermented products [13, 14], rice and rice-based fermented products [13, 14, 136, 140], sweet sorghum [13, 14], wheat [13, 14, 136, 141], rye [13, 14], oat [13, 14], barley [13, 14, 136] and other starchy substrates [134, 137, 142, 143, 144, 145, 146, 147].

#### 3.2.2 Lignocellulosic biomass

**Figure 3:**
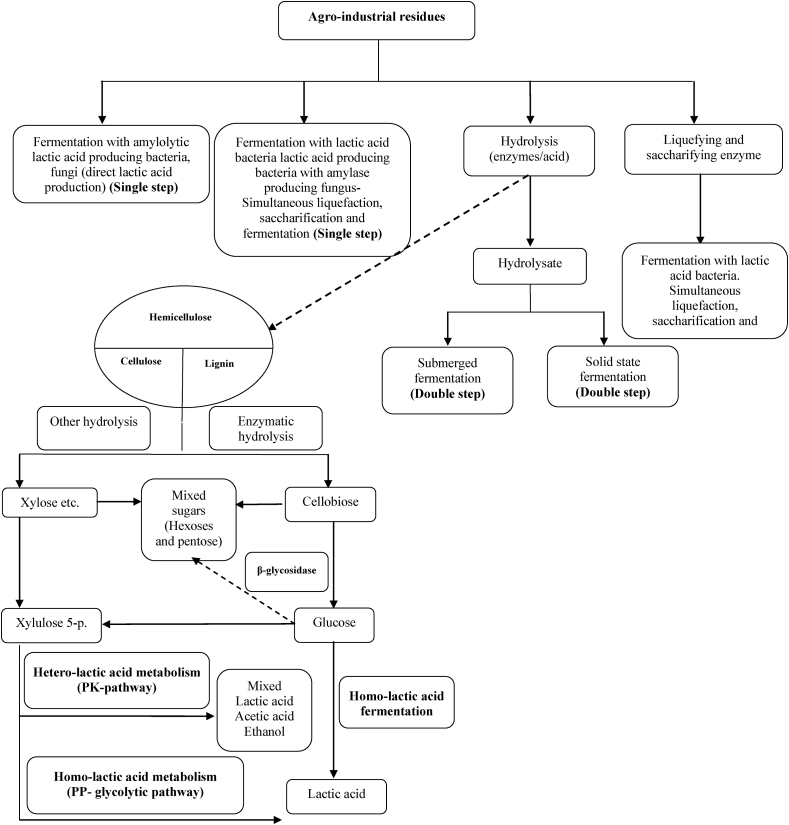
Different modes of fermentative production of lactic acid.

**Figure 4:**
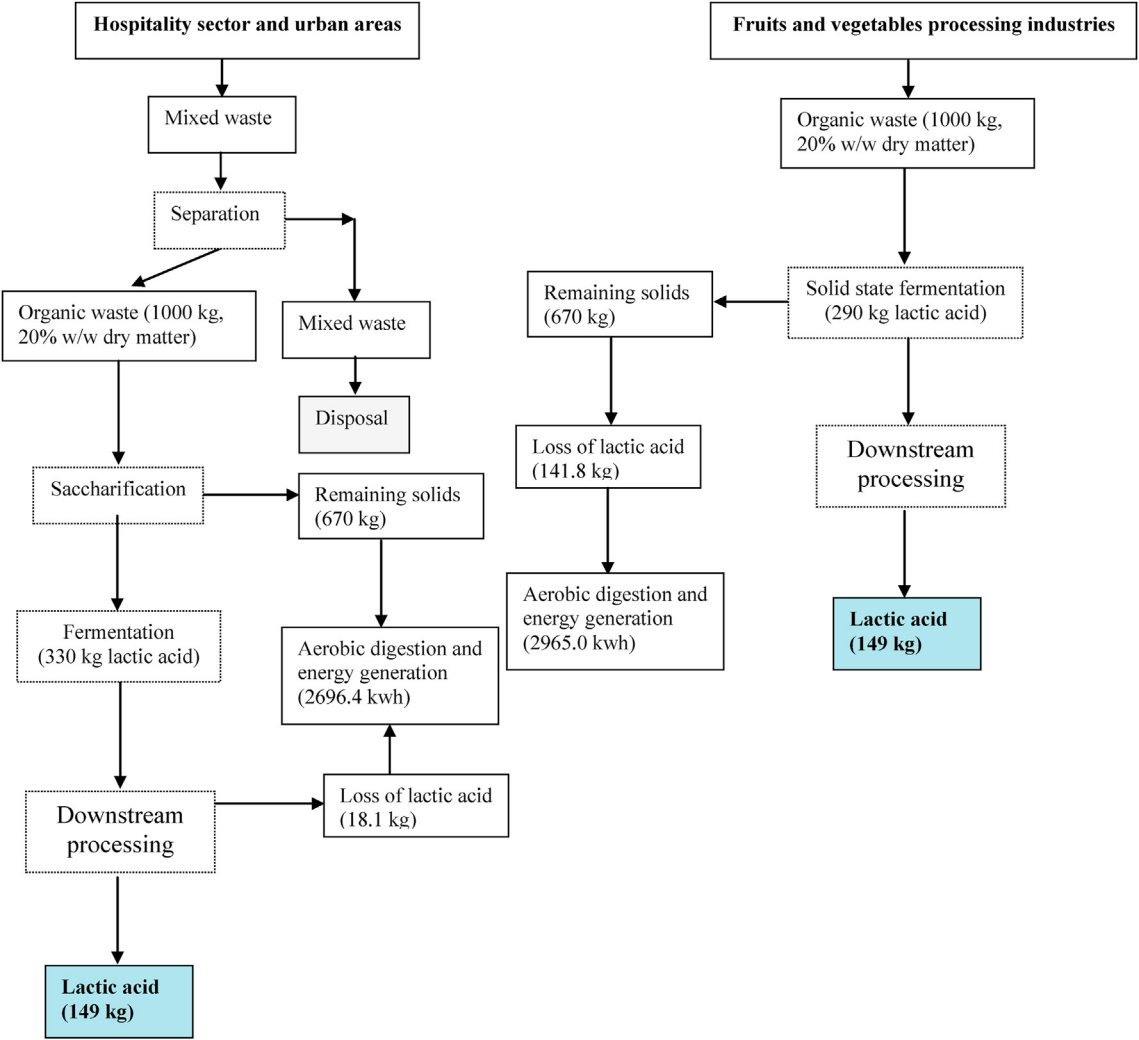
Lactic acid production from urban areas or the hospitality sector, and fruits and vegetables industry (Demichelis et al., 2017).

Worldwide, there are abundant and cheap lignocellulosic materials, that include agricultural residues (corn stover, bagasse, and rice husk), forestry residues (sawdust), portions of municipal solid wastes (waste paper and brewer spent grains), herbs, switch-grass and shrubs (switchgrass and water hyacinth), woody plants (poplar trees), Stems, straws, leaves, stalks, shells, husks, and peels from cereals like wheat, rice, barley, corn, sorghum and various industrial wastes [Figures 3 and 4; [134,148]. Cellulosic materials are composed mainly by cellulose, xylan, arabinan, galactan, and lignin [13, 14, 149].

The addition of pectinases and cellulases in the fermentation medium can enhance LA production [150]. However, fermentation of lignocellulosic hydrolysates is prevented by the inhibitory effect of some compounds including acetic acid, furfural, and 5-hydroxymethylfurfural, which are formed during pre-treatment of lignocellulose [150]. To reduce this inhibition, studies were performed through physical and chemical detoxification of the hydrolysate, being this mentioned as the challenges that must be overcome for their efficient utilization [14]. For LA production, several cellulosic materials can be used as substrate, such as: pure cellulose [13, 14, 151], lignocellulosic pentoses including xylose and arabinose (Figure 2) [13, 14, 63, 65, 66, 152] corncob [63, 65] waste paper [13, 14, 153, 154], and wood [64, 130, 155].

Yadav et al. (2020) indicated that *P. pentosaceus* SKL-7, *Lb. plantarum* SKL-19, *Lb. fallax* SKL-15, *Lb. plantarum* SKL-22, and *Lb. paracasei* SKL-21grew well in presence of 1-Ethyl-3-methylimidazolium-acetate, 1-Butyl-3-methylimidazolium methane-sulfonate and 1-Butyl-3-methylimidazolium chloride. The *L. plantarum* SKL-22 demonstareted relatively high tolerance with greatest specific growth rates in presence of 0.5% and 1% 1-Butyl-3-methylimidazolium methane-sulfonate and 1-Butyl-3- methylimidazolium chloride. *L. plantarum* SKL-22 formed reasonable good content of lactic acid 34.26 g/l, so promising strain for production of lactic acid from lignocellulosic biomass [156].

Agricultural residues are another potential source of substrates for LA production. This category includes: alfalfa fiber [157], wheat bran and straw [158, 159], defatted rice bran [160, 161], food wastes [162], corn stover and cob [29, 65, 152, 157, 163], barley bran husks [163], sugarcane and cassava bagasse [164, 165, 166], trimming vine shoots [163], wine-trimming wastes [163], apple pomace [167], banana wastes [168], mango peel [169], mussel processing wastes [13, 14], cellulosic bio sludge [170], kitchen refuses and wastes [27, 171, 172], fish meal wastes [173], cardboard waste [154] and sugarcane bagasse waste [174]. Wastewater of paper sludges is another source that does not require pretreatment and have a high content of polysaccharide degradation products and short cellulose fibers [20, 68, 170, 175, 176].

### 3.3 Dairy wastes

#### 3.3.1 Cheese whey

Whey is the primary by-product of the dairy industry, containing proteins, lactose, fats, water-soluble vitamins and minerals. Lactose can be hydrolyzed into glucose and galactose by entering the cell via a permease and β-galactosidase (Figure 1) and can produce four LA moles [122, 177]. LAB are fastidious microbes that require complex macro and micronutrients since they don't have enough proteolytic enzyme activity to utilize whey proteins [178]. For complete utilization of whey lactose and proteins, the addition of supplementary components with a nitrogen source such as yeast extract, peptone, and soy flour or steep corn liquor is necessary. Enriched whey showed a significant improvement in LA production [13, 108, 122, 177]. For instance, whey supplemented with whey protein hydrolysate or yeast extract enhanced the LA production and decreased the unused nutrients loss during bioprocessing [178, 179].

Several strains have been used for LA production from whey, including *Lb. plantarum, Lb. helveticus*, *Lb. acidophilus*, *Lb. delbrueckii* subsp*. bulgaricus*, *Lb. casei*, *L. lactis*, and *K. marxianus*. However, in conventional batch fermentation, there is a long lag phase in LA production from whey. To overcome this problem, a greater fermenter capacity is required, but this subsequently increases the operational costs [13, 14, 179]. On the other hand, continuous whey fermentation (without the requirement of high-volume) allowed obtaining a high LA productivity [13, 14, 180]. Semi-continuous fermentation conditions with nanofiltration membranes for recycling lactose and cells increased twice the LA production [181]. *Lactobacillus* and *Lactococcus* genus are the major LA producers who could efficiently utilize lactose and proteins, present in whey, with high conversion rates [13, 14, 179, 182, 183]. *Lb*. sp. RKY2, *Lb. casei* NRRL B-441 and *L. lactis* subsp*. cremoris* produced LA at 6.34, 3.97 and 4.6 g l^−1^ h^−1^; with a yield of 0.98, 0.93 and 0.88 g/g lactose, respectively [13, 14, 182, 184]. Also, *B. longum* NCFB 2259 could produce LA with a yield of 0.81 g/g whey lactose as a sole medium in a batch fermentation reactor [181]. On the other hand, LA initially present in whey could have an inhibitory effect in whey fermentation which can be reduced to a certain content by the application of mono or dipolar membranes in an electrodialysis system [185] or using a hollow fiber fermenter by a continuous dialysis process [13, 14].

#### 3.3.2 Yogurt

There is a huge amount of damaged or expired yogurt as waste products, which could provide a good resource for LA production [186]. Sweetened yogurt contains additional sugars, including sucrose and glucose, which would lead to a higher LA production, in comparison to cheese whey, which has fewer sugars. From yogurt whey LA was obtained with a productivity of 0.76 g/L/h and a yield of 0.9 g/g by *Lb. casei* ATCC 393 with bioconversion of about 44% of total sugars, with increasing order of consumption glucose > sucrose > lactose [186].

### 3.4 Industrial waste

This category includes glycerol from biodiesel industry and petroleum-based polymers. Glycerol is a byproduct of biodiesel industry that can be produced at a weight ratio of 1:10 (glycerol:biodiesel) [187]. There is abundantly glycerol being a cheap raw material that could be utilized by several microorganisms, which can convert glycerol to LA, such as *Klebsiella pneumonia* [188], *Clostridium pasteurianum* [189], *Lb. Reuteri* [13, 14], *Lb. Brevis* [13, 14], *Lb. Buchneri* [13, 14], wild/engineered E. coli [53, 190, 191, 192, 193]. Engineered *Enterococcus faecal* [194]*,* and *Achromobacter denitrificans* NBRC 12669 [195]. According to Mazumdar et al. (2010) [53] and Posada et al. (2012a, b) [59,187,196,197], the over expressing pathways in engineered *E. coli* strains via homofermentative route could convert glycerol to D-lactate [59, 187, 196].

### 3.5 Microalgae

Algal biomass is another source for LA production [15, 108, 134]. Some advantages of these substrates include: 1) the richness in carbohydrates, essential fatty acids, vitamins, and proteins; 2) the lignin absence in microalgae could simplify its conversion into fermentable sugars [198,199]; 3) the growth can be almost anywhere with extremely short harvest cycles of about 1–10 days [197]. 4) The use of microalgae and cyanobacteria is capable to decrease the feedstock cost, as a result of their ability to utilize light energy to fix CO_2_ [134]. The microalga *Hydrodictyon reticulum* has been utilized as a substrate for the production of L-LA by *Lb. paracasei* LA104 and *Lb. coryniformis* subsp*. Torquens* [198]. *Lb. paracasei* LA104 and *L. coryniformis subsp. torquens*, by simultaneous saccharification and co-fermentation*,* achieved values of 37.1 g/l and 36.6 g/l LA and D-LA, respectively, from 80 g *Hydrodictyon reticulatum* (47.5%) [198, 199]. Lipid-free microalgae are good sources for LA production, such as *Nannochloropsis salina* for *Lb. Pentosus* [199], *Chlamydomonas reinhardtii*, *Chlorell pyrenoidosa*, and *Dunaliella tertiolecta* for *L. amylovorus* [13, 14].

### 3.6 Feed stock pretreatment

Generally, three leading stages could be demonstrated for efficient fermentative LA production mainly (i) feedstock pretreatment, (ii) mixed and other substrates for LA production, (iii) ion requirement [10, 134, 147, 200].

The chemical composition of substrate mainly consist of carbon and nitrogen compounds. A lignocellulosic agricultural residue as worldwide resource is comprised of three main polymers: cellulose, hemicellulose and lignin, linked by covalent and non-covalent bonds. Not only, this organised structure cause to prevent cellulose and hemicelluloses hydrolysis into fermentable sugars, but also inhibit the valorisation of lignin into chemicals. The impacts of various pretreatment methods upon diverse lignocellulosic materials, e.g., wheat straw, corn stover, rice straw, switchgrass, and sugarcane bagasse have been demonstrated [10, 14, 134, 147, 200]. The pretreatment process is extremely crucial stage in lignocellulose bioconversion. If it is too intense, toxic compounds can be generated which prevent microbial metabolism and growth. In contrast, insufficient pretreatment will cause, the resultant residue is not easily saccharified through hydrolytic enzymes. Therefore, pretreatment has a great potential to affect the downstream costs due to enzymatic hydrolysis rates, enzyme loading, determining fermentation toxicity, mixing power, power generation, product purification, product concentrations, waste treatment demands, and other process variables. Numerous pretreatments for lignocellulosic materials are suggested as follow:

#### 3.6.1 Physical pretreatment

1) Milling is being conducted for approximately all solid feedstocks to decrease particle size and cause it more accessible to other treatments or hydrolysis.

In order to improve fermentation, hydrolysis of carbohydrates to fermentable sugars is performed to facilitate microorganisms growth and their accessibility for biochemical conversion to LA. The hydrolysis of starchy substrate is carried out by amylolytic enzymes upon gelatinization, liquefaction and saccharification. The optimization of hydrolysis could be conducted for numerous substrates, temperature, time and mixing conditions etc [10, 14, 134, 147, 200]. 2) Liquid hot water and emerging technologies including pulsed electric field, high hydrostatic pressure and high pressure homogenization, ionizing (X-ray, beam) and non-ionizing (microwaves) radiation and non-thermal plasma can be also suitable as pretreatments or co-treatments during hydrolysis in biorefinery processes, predominantly for lignocellulosic substrates and other substrates [10, 14, 134, 147, 200].

#### 3.6.2 Chemical pretreatment

Combination of thermal pretreatments with alkaline, lime, organosolv, ammonia fiber explosion and ammonia recycle percolation, ionic liquid, natural deep eutectic solvents are “greener” method, and acids, making changes in all three portions of lignocellulose substrate [10]. Acid treatment was predominantly applied in the hydrolysis of lignocellulose. Dilute acid pretreatment reaction can cleave labile ester groups and catalyze the hydrolysis of the glycosidic bonds of hemicellulose and lignin. Hydrolysis of both hemicellulose and lignin, in turn, production of toxic by-products. Although, it minimizes the requirement for using hemicellulases, acid hydrolysis cannot be combined with further enzymatic steps. Moreover, thermo-chemical pretreatments are considered as energy demanding and not environment friendly. The major drawback in the production of LA on lignocelluloses is formation of numerous undesirable compounds including furfural, uronic acid, vanillic acid, 4-hydroxybenzoic acid lignin or salts can influence microbial growth during fermentation and slow-down the fermentation and increase purification costs [10, 14, 134, 147, 200].

#### 3.6.3 Biological pretreatments

This category of pretreatment is greater eco-friendly method than others and consists of various methods including:

1) Utilization of more productive species to decline time necessary for microbial growth and formation of enzymes and hence cause to increase efficiently and economically processes. For instance, basidiomycetes or their enzymes (lignin peroxidase, laccase and manganese peroxidase) to degrade lignocellulosic biomass [10, 14, 134, 147, 200].
2) Enzymatic hydrolysis is the abundant method to produce fermentable sugars from pretreated lignocellulosic biomass via depolymerizes the polysaccharides in the water-insoluble solid fraction. Therefore, it is critical step to consume polysaccharides as a carbon source by LAB [14]. Cellulases and hemicellulases enzymes can convert cellulose and hemicellulose into soluble sugars, respectively. In order to enhance enzymatic hydrolysis efficiency, mixtures of these enzymes are required to improve hemicellulose hydrolysis and then rise the access of cellulase, inducing to a reduced hydrolysis time and process cost [14]. Effective degradation and saccharification of cellulose demand a synergistic reaction of the 3 categories of cellulolytic enzymes in order: (i) Endo-β-1,4-glucanases (EG; EC 3.2.1.3) can randomly dissociate accessible intramolecular β -1,4-glucosidic bonds of cellulose chains, generating a new reducing and non-reducing chain end pair. (ii) Exo- β -1,4-glucanases or cellobiohydrolases (CBH; EC 3.2.1.91) can hydrolyze cellulose chains at the ends of the polymer, forming soluble cellobiose or glucose. (iii) β -Glucosidases (β -G; EC 3.2.1.21) (cellobiases) are capable complete the hydrolysis by cleaving cellobiose into 2 glucose molecules. They are also active on cellooligosaccharides. Besides, there are accessory or “helper” enzymes that play a main role in hydrolysis by clearing the access of the leading enzymes to cellulose due to attack hemicellulose and lignin. Xylan does not generate tight crystalline structures, so the substrate is more easily accessible. However, in contrast to cellulose, xylans are chemically quite complex, and their hydrolysis needs multiple enzymes. Enzymatic hydrolysis of hemicellulose was performed by β -xylosidase, endo-1,4- β -xylanase, β - glucuronidase, α -l-arabinofuranosidase, galactomannanase, glucomannanase and acetylxylan esterase, which act on xylan cleavage and saccharification. β -mannanase and β -mannosidase, which cause to cleave the glucomannan polymer backbone [14]. The hydrolytic efficiency of lignocellulose substantially improved by utilizing combinations of the 3 enzymes, 2 cellulases, and 1 xylanase [10, 14, 134, 147, 200].

#### 3.6.4 Mixed and other substrates for LA production

Wastes or by-products are main representatives of mixed substrates with different composition of carbohydrates and proteins. Meanwhile, they contain low nutritional values, so require additional fortification and often some treatment. Inhibitory or toxic components in these media have to be evaluated, also. Instead of consumption yeast extract or other Unconventional and expensive nitrogen sources, numerous agricultural residues or byproducts namely soya bean hydrolysate, corn steep liquor, corn meal and wheat bran hydrolysate, chicken feather hydrolysate, by-products from malting and brewing and oil production can be utilized as cheaper nitrogen sources [10, 14, 134, 147, 200] (Table 1). Substantial studies were demonstrated in the case of free amino nitrogen content such as amino acids, and phosphate to LA production. Complementary substrates in nitrogen and carbohydrate sources were combined for LA fermentation. For instance sugar beet molasses (rich in carbohydrates) and distillery stillage from bioethanol production from waste potato (rich in nitrogen source) were used for LA production by *Lactobacillus paracasei*. Many studies have shown that how to determine carbon to nitrogen ratio and correlate it with LA productivity. Carbon/nitrogen ratio significantly effects on LA yield and cell growth. When the carbon and nitrogen content are provided only from fermentable sugars and free amino nitrogen content, accurate optimization of media composition for LA production would be performed [10, 14, 134, 147, 200].

#### 3.6.5 Ion requirement

It is obvious that metals play a key role in the biological processes, such as activating major enzymes in metabolisms as cofactor, improving the growth of microbial cells and activation of organic acid synthesis by fungal and bacterial species [201].

##### 3.6.5.1 Copper

Copper (II) by far has acted as a cofactor within numerous copper-dependent enzymes [201]. Furthermore, the microbial populations including LAB are more affected in the presence of copper (II) [202, 203, 204]. There are several hypotheses to improve lactic acid production in the presence of copper: a) it was proved that copper (II) inhibited the conversion of D-lactic acid to pyruvate via preventing the activity of NAD independent D-lactate dehydrogenas (id-LDH) in the pure culture, b) carbohydrate hydrolysis and glycolysis pathway were both strengthened that resulted in the promoting of lactic acid production from organic waste. The amount of copper (Cu-15; 15 μM/g, Cu-30; 30 μM/g and Cu-70; 70 μM/g) influence on the production of lactic acid (23.21 g/L), (17.44 g/L) and (16.53 g/L), respectively. It is indicated that the maximum concentration of lactic acid increased in the presence of copper compared to that of Blank (13.11 g/L). Nevertheless, continuously raising the copper level gradually reduced the production of lactic acid imply that that 70 μM-Cu^2+^/g VSS might exceed the tolerance of Lactobacillus and variation of functional genes revealed that the suggested homeostatic system towards copper (II) was activated at pretty low content that cause to facilitate the membrane transport function as well as carbohydrate metabolism [201].

##### 3.6.5.2 Zinc

Regarding to Mumtaz et al., 2019, ZnO solubilization was associated to the synthesis of specific organic acids like Lactic and acetic acids. The culture medium was acidified and then ZnO solubilized. Two Zn- and acid-tolerant strains. Rhizosphere isolate Bacillus sp. ZM20 and B. cereus culture-collection strain generated various organic acids at a remarkably greater content than less tolerant strains when cultured in the presence of inhibitory but non-lethal levels of ZnO. It is supposed that the enhanced synthesis of these acids is due to a generalized stress response [205].

## 4 Conclusions

The capacity of several microorganisms for production of LA was studied. Some of these microorganisms such as LAB require complex nutrients and low fermentation temperatures, which lead to increased costs and contamination risk. However, some of them like *Bacillus* spp., reduce the LA production cost due to fewer nutrition demands and a high temperature of fermentation. Agro-industrial waste or sub-products with a lower value such as molasses, juices waste, starchy biomass, agricultural residues, forestry residues that are rich in mono and disaccharides, which in some cases need to be hydrolysed by pectinases to enhanced the LA production. To use dairy wastes as a substrate, mainly whey, it is necessary to use an enriched mediums, due to insufficient proteolytic enzyme activity.

## Declarations

### Author contribution statement

All authors listed have significantly contributed to the development and the writing of this article.

### Funding statement

This research did not receive any specific grant from funding agencies in the public, commercial, or not-for-profit sectors.

### Competing interest statement

The authors declare no conflict of interest.

### Additional information

No additional information is available for this paper.
